# Roles of Mono- and Bi-articular Muscles in Human Limbs: Two-joint Link Model and Applications

**DOI:** 10.1093/iob/obac042

**Published:** 2022-11-22

**Authors:** Tsutomu Miyake, Masataka Okabe

**Affiliations:** Department of Anatomy, The Jikei University School of Medicine, Tokyo 105-8461, Japan; Department of Anatomy, The Jikei University School of Medicine, Tokyo 105-8461, Japan

## Abstract

We review the two-joint link model of mono- and bi-articular muscles in the human branchium and thigh for applications related to biomechanical studies of tetrapod locomotion including gait analyses of humans and non-human tetrapods. This model has been proposed to elucidate functional roles of human mono- and bi-articular muscles by analyzing human limb movements biomechanically and testing the results both theoretically and mechanically using robotic arms and legs. However, the model has not yet been applied to biomechanical studies of tetrapod locomotion, in part since it was established based mainly on mechanical engineering analyses and because it has been applied mostly to robotics, fields of mechanical engineering, and to rehabilitation sciences. When we discovered and published the identical pairs of mono- and bi-articular muscles in pectoral fins of the coelacanth fish *Latimeria chalumnae* to those of humans, we recognized the significant roles of mono- and bi-articular muscles in evolution of tetrapod limbs from paired fins and tetrapod limb locomotion. Therefore, we have been reviewing the theoretical background and mechanical parameters of the model in order to analyze functional roles of mono- and bi-articular muscles in tetrapod limb locomotion. Herein, we present re-defined biological parameters including 3 axes among 3 joints of forelimbs or hindlimbs that the model has formulated and provide biological and analytical tools and examples to facilitate applicable power of the model to our on-going gait analyses of humans and tetrapods.

## Japanese

本総説は、ヒトやヒト以外の四肢動物における歩容解析を含む四肢運動を生体工学研究へ応用することを目的として、ヒトの上腕や大腿の一関節筋・二関節筋リンクモデルを概説したものである。このモデルは元来、ヒトの四肢運動から得られた生体力学的解析結果を、ロボットアームやロボットレッグを用いて理論的・力学的に検証するというアプローチで提唱された概念である。機械工学的な解析に基づいて確立された研究背景から、このモデルは主に、ロボット工学や機械工学、リハビリテーション科学の分野への応用が進んだが、四肢動物の四肢運動に関する生体力学への応用は進まなかった。同じ頃、我々は、シーラカンス*Latimeria chalumnae*の胸鰭にヒト上腕と相当の対をなす一関節筋・二関節筋が存在することを発見し、鰭から四肢への進化や四肢運動において、これらの筋が重要な役割を果たす可能性を見出していた。この発見を起点として、我々は、四肢動物の四肢運動における一関節筋・二関節筋の機能的役割を明らかにするために、このモデルの理論的背景や機械的パラメーターを再検証した。その結果、このモデルが定式化した前肢や後肢の三関節間にまたがる三つの軸を含むパラメーターを補正し、生物学的パラーメーターとして再定義することで、四肢動物の四肢運動の解析が可能となるという結論に至った。本総説では、我々が現在進行中の歩容解析を例に、ヒトやヒト以外の四肢動物における四肢運動の生体工学研究へこのモデルを応用するための生物学的解析手法を含めて概説する。

## Introduction

We stand, walk, and run. In fact, we take for granted that we can do these things and rarely think about how we actually can do all. We thus imagine how difficult it has been for robotic engineers to build the first tetrapod-like limbs that can stand, walk, and/or run. Because of widely accepted views of biomechanics and human anatomy and their associated historical conventions, almost all robots that have been built included an actuator(s) in a joint. Under the control of a sophisticated computer-program(s), robots are able to stand, walk, and/or run. The theory behind all such efforts to date is the joint-torque control model (Geyer and Herr 2010; Agbarajl et al. 2017). This idea has inspired our imagination, but it does not appear to be realistic. As far as we know, no extinct and extant tetrapods including humans have ever evolved an actuator in any limb joints. There have been a few robots with their limbs equipped with muscle-like robotic parts, since a number of technical hurdles that have prevented engineers from utilizing muscle-like parts for robotic limbs have existed (Biswal and Mohanty 2021).

The muscles of limbs also have their own issues including a paradox that has been debated in biomechanical science (van Ingen Schenau et al. 1987; van Ingen Schenau 1989). In our hindlimbs and forelimbs, there is a pair of muscles that cross two joints in series, called bi-articular muscles. When the rectus femoris contracts in our thigh, it flexes the hip joint but extends the knee joint. The biceps femoris long head extends the hip joint but flexes the knee joint. The phenomenon has been called Lombard's paradox (Lombard 1903; van Ingen Schenau 1990). However, during a running, both the hip joint and the knee joint extend when one heel touches the ground. Furthermore, as a person jumps straight up, both the joints extend as well. van Ingen Schenau (1990) reviewed functional roles of bi-articular muscles in limbs and introduced his theme of external forces and net joint moments based on inverse dynamical analysis. He then stated that “the realization of an external force of certain direction and magnitude always requires the control of a distinct combination of net moment.” Furthermore, he introduced the control of position and force by illustrating two types of transformation: (1) muscle displacement to joint rotations to hand/foot displacement and (2) muscle force to joint moments to external force. In his review, the two transformations are not symmetrical, indicating that the two transformations are not necessarily made in a one-to-one correlation, but that they do conflict when we consider two joints, each of which is crossed by an antagonistic pair of bi-articular muscles. The bi-articular muscles between the shoulder joint and the elbow joint, which were discussed by van Ingen Schenau (1990), serve as one such example.

**Fig. 1:**
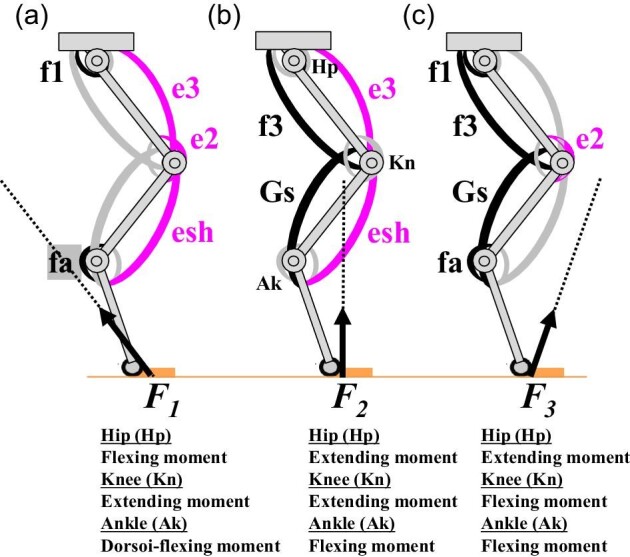
Differential activations of mono- and bi-articular muscles under three ground reaction forces (*F_1_*, *F_2_*, *F_3_*) when the hindlimb is extended. van Ingen Schenau (1990) illustrated this “possible design” of a hindlimb with mono- and bi-articular muscles based on his review of functional roles of bi-articular muscles in limbs. Only activated muscles are labeled in each case. (**A**) *F_1_* is behind the ankle (Ak) joint; (**B**) *F_2_* is in front of the hip (Hp) joint and the ankle (Ak) joint but behind the knee (Kn) joint, as in humans; (**C**) *F_3_* is in front of the knee joint (Kn), as in most tetrapods. f1: a mono-articular muscle of the hip joint; e2: a mono-articular muscle of the knee joint; e3 and f3: bi-articular muscles of the thigh; Gs: gastrocnemius; fa: a mono-articular muscle of the ankle joint; esh: a bi-articular muscle of the lower leg other than Gs. Humans do not have esh. The rostral direction of the body is on the right side.

Through his analysis of bi-articular muscles (van Ingen Schenau et al. 1987) and his view of multi-joint movements (van Ingen Schenau 1989), van Ingen Schenau (1990) has focused on the important concepts that our present review discusses below (Fig. 1). These include the joint link segment; forces and directions that a pair of bi-articular muscles exerts and determines; the coordination of mono- and bi-articular muscles; co-activation of an antagonistic pair of muscles; control position and force at the distal end of the link; and ground reaction forces. van Ingen Schenau(1990) proposed the “possible design” of limb muscles in light of these concepts and discussed possible activation patterns for a different set of mono- and bi-articular muscles under three different ground reaction forces (Fig. 1). Later, Bolhuis et al. (1998) examined the activation patterns of mono- and bi-articular muscles in human forelimbs; they found a different set of activation patterns between mono- and bi-articular muscles.

By the time that van Ingen Schenau's review (van Ingen Schenau 1990) was published, a Japanese group had begun their research to elucidate functional roles of mono- and bi-articular muscles in human limbs by analyzing limb movements biomechanically and testing the results theoretically and mechanically using robotic arm and leg models (Fujikawa et al. 1996; Fujikawa et al. 1997; Oshima et al. 1999; Fujikawa et al. 2001; Kanayama et al. 2001; Oshima et al. 2001). These studies introduced the two-joint link model of mono- and bi-articular muscles in human limbs by a series of theoretical and mechanical engineering concepts: the two-joint link; three defined axes of the brachium or thigh; output forces and directions that a different combination of mono- and bi-articular muscles exert; the coordinated activities of mono- and bi-articular muscles; activity switches between a different antagonistic pair of mono- or bi-articular muscles; a control position and force at the wrist or the ankle; and contact task.

The above Dutch and Japanese group have thus independently proceeded their own studies of the biomechanics of human limb locomotion, and the Dutch group shared a number of underlying concepts for the functional roles of bi-articular muscles along with mono-articular muscles in linked joints of the limbs with those of the Japanese group (Bobbert and van Ingen Schenau 1988; van Ingen Schenau et al. 1994; Doorenbosch and van Ingen Schenau 1995; van Ingen Schenau et al. 1995). Unfortunately, the Dutch group did not appear to establish their own model based on their publications, probably because Dr. van Ingen Schenau passed away in 1998 (Bobbert et al. 1998). As the Japanese group has published their core results in Japanese in Japanese journals which have somewhat restricted the subsequent application of their findings, the analyses of Japanese studies have established the two-joint link model of mono- and bi-articular muscles in human limbs based on mathematical and computational modeling and mechanical engineering tests. Therefore, the model has been applied to rehabilitation sciences, robotics, and the fields of mechanical engineering, for example, a development of an externally powered lower limb orthosis (Saito et al. 2005) and a development of a rehabilitation support robot (Tsuji et al. 2014), a development of a lancelet robot (Tsuji 2010), a development of a robotic arm (Salvucci et al. 2011), a development of a twin-lever steering system for vehicles (Tajima et al. 2010; Tajima et al. 2011).

On the other hand, because of mathematical and computational modeling for complex motor controls over bi- and mono-articular muscles, the model has not been applied to biomechanical studies of tetrapods other than humans. When we discovered the identical pairs of mono- and bi-articular muscles in pectoral fins of the coelacanth fish *Latimeria chalumnae* (Miyake et al. 2016) to those of humans that the above Japanese studies have defined, we recognized the significant roles of mono- and bi-articular muscles in evolution of tetrapod limbs from paired fins of sarcopterygian fishes (Amemiya et al. 2013) and tetrapod limb locomotion (Miyake et al. 2016). To investigate further the modeling, mechanically tested results and biological implications of the two-joint link model, we have examined most of the published results, examined mechanical and analytical parameters and built biologically testable parameters and framework of the model. The purpose of this present review is thus to present our results, one model of gait analyses and two examples and to discuss the issues to be addressed for our on-going investigation of gait analyses in human and non-human tetrapods and for future studies of evolution of tetrapod limb locomotion. Our review is inspired by one review publication entitled robotics-inspired biology that Gravish and Lauder (2018) published. The robotics-inspired biology provides biologists with an opportunity to build a hypothesis of biological system that cannot be tested directly using animal models, test the hypothesis using physical models, and re-apply the results to biological systems.

## Biomechanical and mechanical analyses of human limb movements

**Fig. 2:**
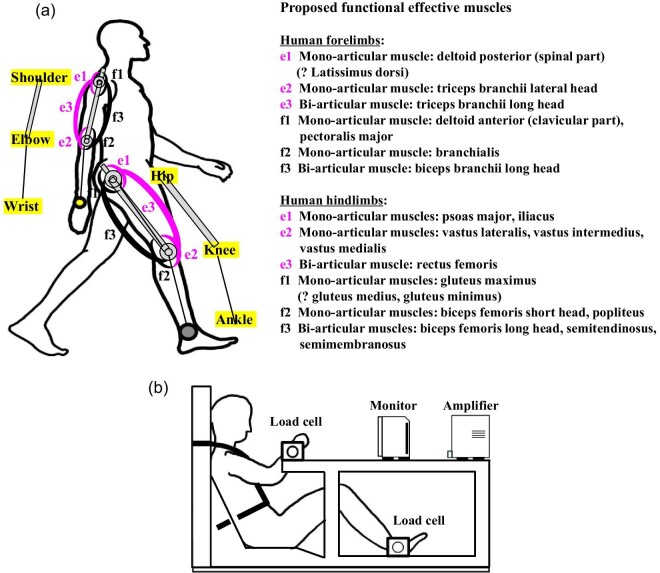
Proposed setups of the two-joint link model of mono- and bi-articular muscles in the human brachium and thigh. (**A**) The functional effective muscles (e-series and f-series) in the human forelimb and hindlimb. (**B**) The experimental platform that has been used to analyze defined movements of either the human forelimb or hindlimb. Designated as e-series and f-series, the muscles consist of two antagonistic pairs of mono-articular muscles (e1, e2, f1, f2) and one antagonistic pair of bi-articular muscles (e3, f3). The activities of the muscles in the link model were hypothesized to generate output forces and thus control directions at the wrist or ankle. Whether the latissimus dorsi should be defined as an e1-series muscle and whether the gluteus medius and gluteus minimus should be defined as f1-series muscles remain to be investigated. The experimental platform was used to analyze defined movements of either the human forelimb or hindlimb. Activities of the functional effective muscles and the maximum output forces were recorded with EMG and a load cell at the wrist or ankle, respectively, (Fujikawa et al. 1996; Fujikawa et al. 1997; Oshima et al. 1999).

**Fig. 3:**
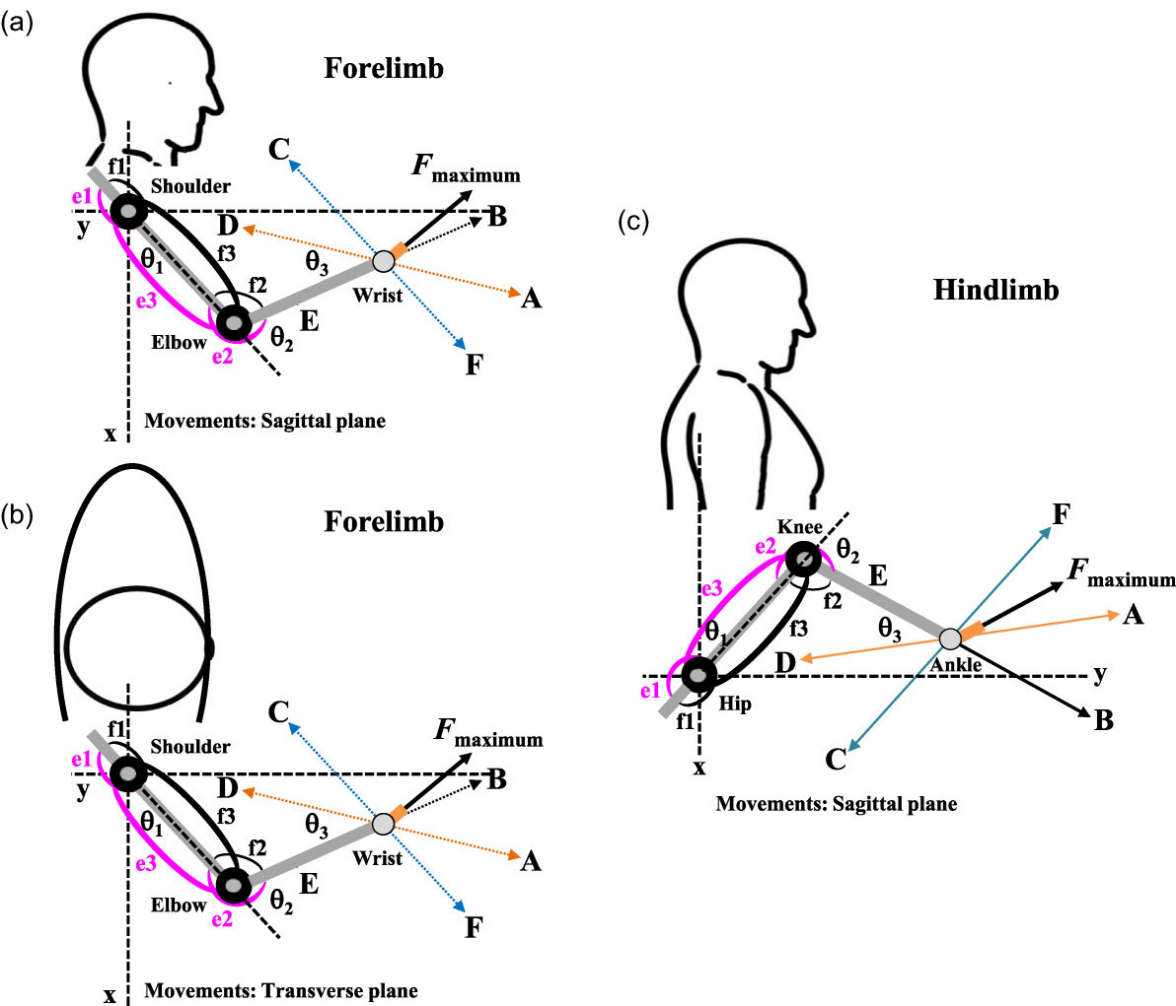
Experimental setups of the two-joint link model for defined movements of the forelimb or hindlimb on a platform. Robotic arms and legs with mono- and bi-articular actuators identical to the muscles of humans were built to test the results of human subjects and establish the mechanical models. (**A**) The defined movements of the forelimb in the sagittal plane (Fujikawa et al. 1996); (**B**) The defined movements of the forelimb in the transverse plane (Fujikawa et al. 1997); (**C**) The defined movements of the hindlimb in the sagittal plane (Oshima et al. 1999; Oshima 2008). θ_1_: the shoulder flexion angle or the hip flexion angle; θ_2_: the elbow flexion angle or the knee flexion angle; θ_3_: the angle between the force direction of A and D and that of B and E; *F_maximum_*_:_ the possible maximum output force exerted by a different combination of mono- and bi-articular muscles in the brachium or the thigh. A–D: the line connecting the shoulder joint and the wrist joint or the hip joint and the ankle joint; B–E: the line connecting the elbow joint and the wrist joint or the knee joint and the ankle joint; C–F: the line parallel to the line between the shoulder joint and the elbow joint or the line between the hip joint and the knee joint.

To investigate functional roles of mono- and bi-articular muscles in the human brachium and thigh, the Japanese group defined the muscles associated with the brachium and thigh as functional effective muscles and designated them as e-series and f-series muscles in the two-joint link model. The anatomical side associated with the e-series and f-series muscles differs between the brachium and the thigh: e-series muscles reside on the dorsal side of the brachium or the thigh whereas f-series muscles reside on the ventral sides of the brachium or the thigh. The muscles designated as e1 and f1 are mono-articular muscles, for example, deltoid posterior and deltoid anterior, respectively, on the shoulder joint and, for example, the psoas major and gluteus maximus, respectively, on the hip joint (Fig. 2A). The muscles designated as e2 and f2 are mono-articular muscles, for example, triceps brachii lateral head and brachialis, respectively, on the elbow joint and, for example, vastus lateralis and biceps femoris short head, respectively, on the knee joint. The muscles designated as e3 and f3 are bi-articular muscles, for example, triceps brachii long head and biceps brachii long head, respectively, crossing between the shoulder joint and elbow joint and, for example, the rectus femoris and biceps femoris long head, respectively, crossing between the hip joint and the knee joint (Fig. 2A; Fujikawa et al. 1996; Fujikawa et al. 1997; Oshima et al. 1999; Oshima et al. 2000; Fujikawa et al. 2001; Oshima et al. 2001; Oshima 2008). In a series of experiments, a human subject on a platform was safely secured and performed isometric push and pull movements of the forelimb or hindlimb in six different ranges (Figs. 2B and 3). In total, five male subjects for the forelimb and seven male subjects for the hindlimb performed such movements (Fujikawa et al. 1996; Oshima et al. 1999; Oshima 2008). For each movement, electromyographic (EMG) recordings of the muscles were made using surface electrodes and the maximum forces were recorded using a load cell at the wrist or the ankle (Fig. 2B; Fujikawa et al. 1996; Oshima et al. 1999). The psoas major and iliacus, which are designated as e1 series muscles, were not recorded, as they did not appear to be accessible for EMG recordings (Oshima et al. 1999).

Each subject was instructed to make defined isometric pull and push movements of the entire forelimb or hindlimb with maximum force in six different ranges along three axes (A–D, B–E, and C–F, as described below) (Fig. 3). For the forelimb, the defined movements were made in both the sagittal plane (Fig. 3A) and the transverse plane (Fig. 3B) with defined angles (Fujikawa et al. 1997). For the hindlimb, the defined movements were made in the sagittal plane with defined angles (Fig. 3C; Oshima et al. 1999; Oshima 2008). For the purpose of these experiments with human subjects and subsequent testing using mechanical engineering robotic arm or leg models, the 360-degree perimeter of the wrist or the ankle was divided into six ranges, A to F, representing the direction of output forces derived from the defined movements made by the human subjects (Fig. 3). The axis represented by A–D is the line connecting the shoulder joint and the wrist joint or connecting the hip joint and the ankle joint. This axis represents the direction in which mono-articular muscles (e2 and f2) exert their forces. The axis B–E is the line connecting the elbow joint and the wrist joint or connecting the knee joint and the ankle joint. This axis represents the direction in which the mono-articular muscles (e1 and f1) exert their forces. The axis C–F is the line parallel to the line between the shoulder joint and the elbow joint or between the hip joint and the knee joint. This axis represents the direction in which the bi-articular muscles (e3 and f3) exert their forces (Fujikawa et al. 1997; Oshima et al. 1999; Oshima et al. 2000).

In addition, to control the posture of each subject, all experiments were performed with defined angles for the limb joints: θ_1_ (the angle of shoulder flexion or angle of hip flexion), θ_2_ (the external angle of elbow flexion or external angle of knee flexion; the angle between axes BE and CF), and θ_3_ (the angle between axes AD and BE) (Fig. 3). All angles were measured in real time using a non-contact position measuring device (Quick MAG). Different horizontal and vertical directions for the defined movements of the forelimb were generated by changing θ_1_,θ_2_ and θ_3_ in the sagittal plane (Fig. 3; Fujikawa et al. 1996; Oshima et al. 1999A) or by changing θ_1_, θ_2_ and θ_3_ in the transverse plane (Fujikawa et al. 1996; Fig. 3B). Different horizontal and vertical directions for the defined movements of the hindlimb were generated by changing θ_1_, θ_2_ , and θ_3_ in the transverse plane (Oshima et al. 1999; Fig. 3C). The angles were also used to model the robotic arms and legs and analyze properties of the models including calculations of output forces, stiffness control, and trajectory control (Fujikawa et al. 1996; Fujikawa et al. 1997; Fujikawa et al. 2001; Oshima et al. 2001).

## Analyses for the proposal of the two-joint link model

**Fig. 4:**
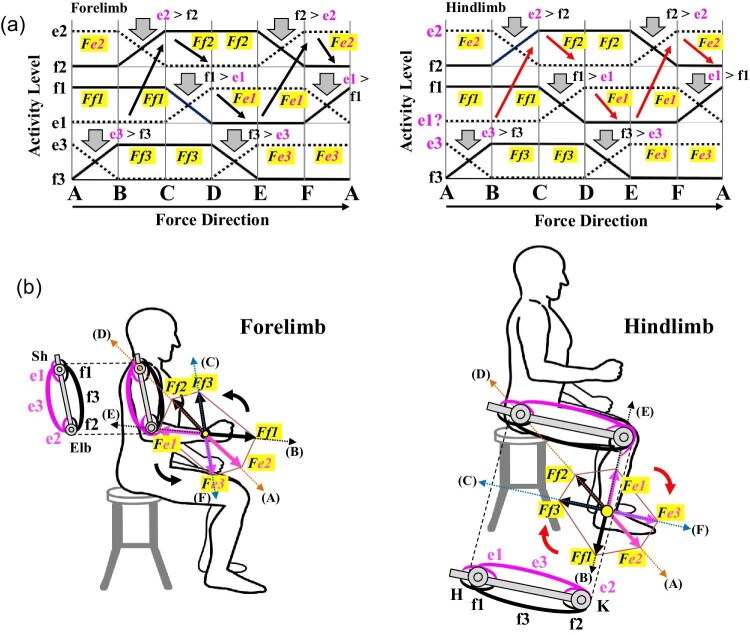
Results of experimentally defined movements of forelimb and hindlimb with EMG analyses. (**A**) Schematized IEMGs based on the EMG data. IEMG showed the maximum output forces and changes in activity between a different antagonistic pair of mono- or bi-articular muscles when human subjects engaged in different defined movements of the forelimb or hindlimb (Fujikawa et al. 1997). The posture condition for the defined movements of the forelimb was set up with θ _1_ = 48° and θ _2_ = 90° (Fujikawa et al. 1997) whereas that of hindlimb was set up with θ _1_ = 45° and θ _2_ = 90° (Oshima 2008). The force directions were normalized to allow visualization of switches in the output force directions. (**B**) A summary of defined movements of the forelimb and hindlimb and the combined distribution of output forces (*Fe1* through *Ff3*) around the 360-degree perimeter of the wrist or ankle. The combined distribution of all output forces becomes hexagonal in the forelimb and hindlimb. Sequential switches of the forelimb are counterclockwise, whereas sequential switches of the hindlimb are clockwise.

**Fig. 5:**
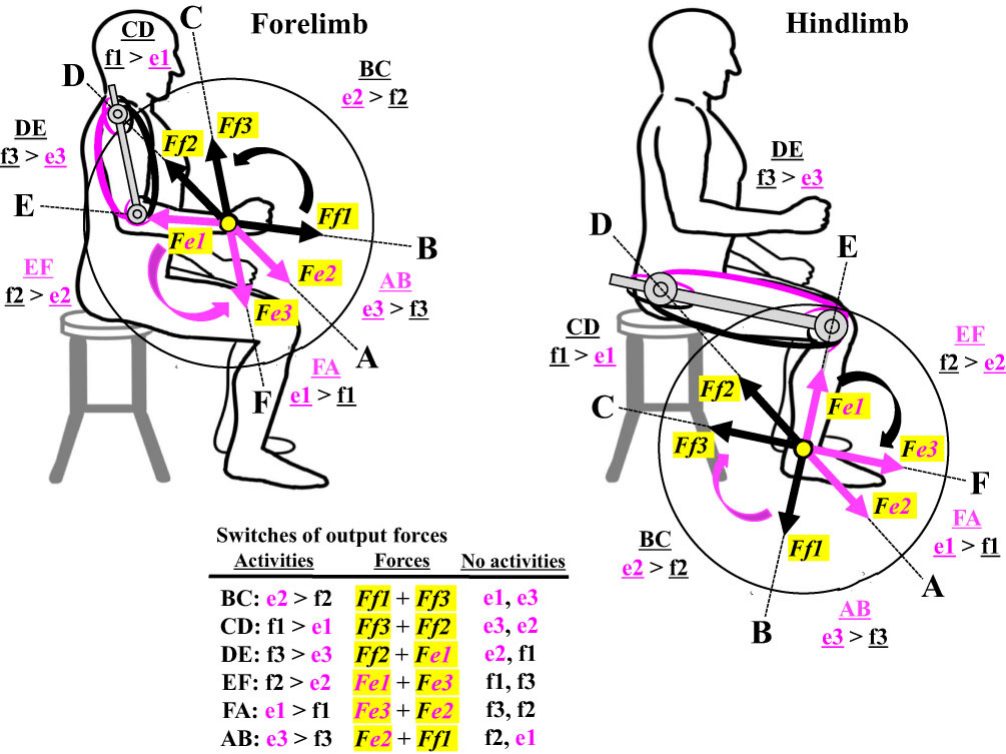
The model of coordinated activities of mono- and bi-articular muscles in the two-joint link model of the human brachium and thigh. The model proposes that switching activity between e1 and f1, e3 and f3, e2 and f2, and forces *Fe1* through *Ff3* occur sequentially around the 360-degree perimeter of the wrist and ankle. For each range, for example, A and B, two muscles (e2 and f1) exert a combined output force (*Fe2* + *Ff1*) with the activity switch from e3 to f3, depending on the combined output force at the wrist or the ankle. Two mono- and bi-articular muscles are inactivated sequentially in each range of activity switches. Sequential switches of the forelimb are counterclockwise, whereas sequential switches of the hindlimb are clockwise.

According to the original figure caption for Fig. 5 (Fujikawa et al. 1996), the electromyograms (EMGs) of around 1.5 s at the maximum output have been combined and arranged to depict an EMG for forelimb movements in the sagittal plane (Fig. 3A). This means that all EMGs associated with e2, f2, f1, e1, e3, f3 over a 1.5-s period at which the maximum force was obtained were assembled based on sequential changes in θ_1_, θ_2_, and θ_3._Fujikawa et al. (1997) subsequently performed similar experiments of forelimb movements in the transverse plane (Fig. 3B) and published their Fig. 6 with the English caption “Raw EMG pattern with changes in the force direction (posture condition: θ_1 _= 48^0^, θ_2 _= 90^0^).” This time, they showed the EMG with one posture condition. In addition, they produced their integrated electromyography (IEMG) data using the data from seven human subjects (Fujikawa et al. 1997, Fig. 7) to clarify trends in muscle activities of all seven human subjects. In addition, the intervals for each of the five force directions, AB, BC, CD, DE, and EF, were all set to be equivalent because of changes in their postures. Their IEMGs were then shown for all postures in all seven human subjects (Fujikawa et al. 1997). Fig. 4A shows a schematized IEMG for the forelimb (Fujikawa et al. 1997) and for the hindlimb (Oshima et al. 1999; Oshima 2008). As the force directions changed from A through F, a different set of mono- and bi-articular muscles exerted their output forces with a switch of activities between an antagonistic pair of mono- or bi-articular muscles (between e2 and f2, f1 and e1, or e3 and f3). As described below, in each switch, AB through FA, a defined combination of one mono- and one bi-articular muscles, therefore, exerts the output force to each defined range and the combined force determines the force direction (Fig. 4B).

Due to limitations of testing them directly, the results of the EMGs and IEMGs have subsequently been analyzed mathematically and mechanically to examine the control of output forces, relationships between the maximum output forces and output forces, stiffness and trajectory controls by adopting the two-joint link model with mechanical robotic arms or legs equipped with or without mono- and/or bi-articular actuators (Fujikawa et al. 1996; Fujikawa et al. 1997; Oshima et al. 1999; Oshima et al. 2000; Oshima et al. 2001; Fujikawa et al. 2001; Oshima 2008). These physical models allowed them to test, for instance, what would happen to output forces and directions and contact task if either a mono-articular muscle, a bi-articular muscle, or a combination of mono- and bi-articular muscles are absent. The findings and conclusions from these studies are briefly summarized and illustrated in Figs. 4 and 5.

According to these studies, the defined movements of forelimbs or hindlimbs across the six different ranges, A through F, produced a definitive pattern of muscle activities (Fig. 4A). For instance, in A and B, antagonistic muscles (e3 and f3) switch their activities, with one being turned off (e3) whereas two of the remaining mono- and bi-articular muscles (e2 and f1) exert their output forces (*Fe2 *+ *Ff1*) as a combined force. The combined force will determine the force direction, since the output force and direction of combined forces are characterized to be a vector quantity consisting of force and direction (Newton's second law) (Caldwell et al. 2014). As the force moves from A through F, a different antagonistic pair of mono- or bi-articular muscles switches the activities sequentially, while two of the remaining mono- and bi-articular muscles produce the output forces at the wrist or the ankle. The sequential switches differ between forelimb and hindlimb in being counterclockwise and clockwise, respectively, (Figs. 4B and 5). Due to the sequential switches, two of the mono- and bi-articular muscles will be inactivated sequentially for each range (Fig. 5). If an inactivated muscle happens to become activated in a given range, it might be regarded as an independent activation of the two-joint link model. To date, these studies have verified this definitive pattern of activation switches using mechanical robotic models and proposed the hexagonal distribution of all combined forces and directions in the ranges A through F (Fig. 4B; Fujikawa et al. 1996; Fujikawa et al. 1997; Oshima et al. 1999; Oshima et al. 2000; Oshima et al. 2001; Fujikawa et al. 2001; Oshima 2008).

When the entire forelimb or hindlimb moves, as described above, an antagonistic pair of mono- or bi-articular muscles switches their activities and two of the remaining mono- and bi-articular muscles exert their output forces at the wrist or the ankle (Figs. 4 and 5). However, the sequential switches of the forelimb are counterclockwise, whereas those of the hindlimb are clockwise (Figs. 4B and 5). Coordinated activities of mono- and bi-articular muscles of the brachium or thigh have thus been proposed to act on the wrist joint or the ankle joint and control movements of the entire forelimb or hindlimb, respectively, (Fujikawa et al. 1996, Fujikawa et al. 1997; Oshima et al. 1999). Whichever direction each limb moves to, a different set of mono- and bi-articular muscles coordinately exerts forces at the wrist or the ankle and thus controls the force direction. Axis A–D represents the direction in which the mono-articular muscles (e2 and f2) exert their forces. Axis B–E represents the direction in which the mono-articular muscles (e1 and f1) exert their forces. Axis C–F represents the direction in which the bi-articular muscles (e3 and f3) exert their forces (Fig. 5). Each range is thus defined by two axes in both forelimb and hindlimb: AB by axes A–D and B–E, BC by axes B–E and C–F, CD by axes C–F and A–D, DE by axes A–D and B–E, EF by axes B–E and C–F and AF by axes F–C and A–D (Fig. 5). Although the intervals of six ranges were set to be equivalent in the controlled experiments of the two-joint link model (Fujikawa et al. 1997), the actual angles of the ranges will vary considerably, as illustrated later in human sprint and bi-pedal walking gaits, depending on different tetrapod animals, different individuals, their different postures or strides and/or their different activities of limb locomotion.

## Biological prerequisites for application of the two-joint link model

**Fig. 6:**
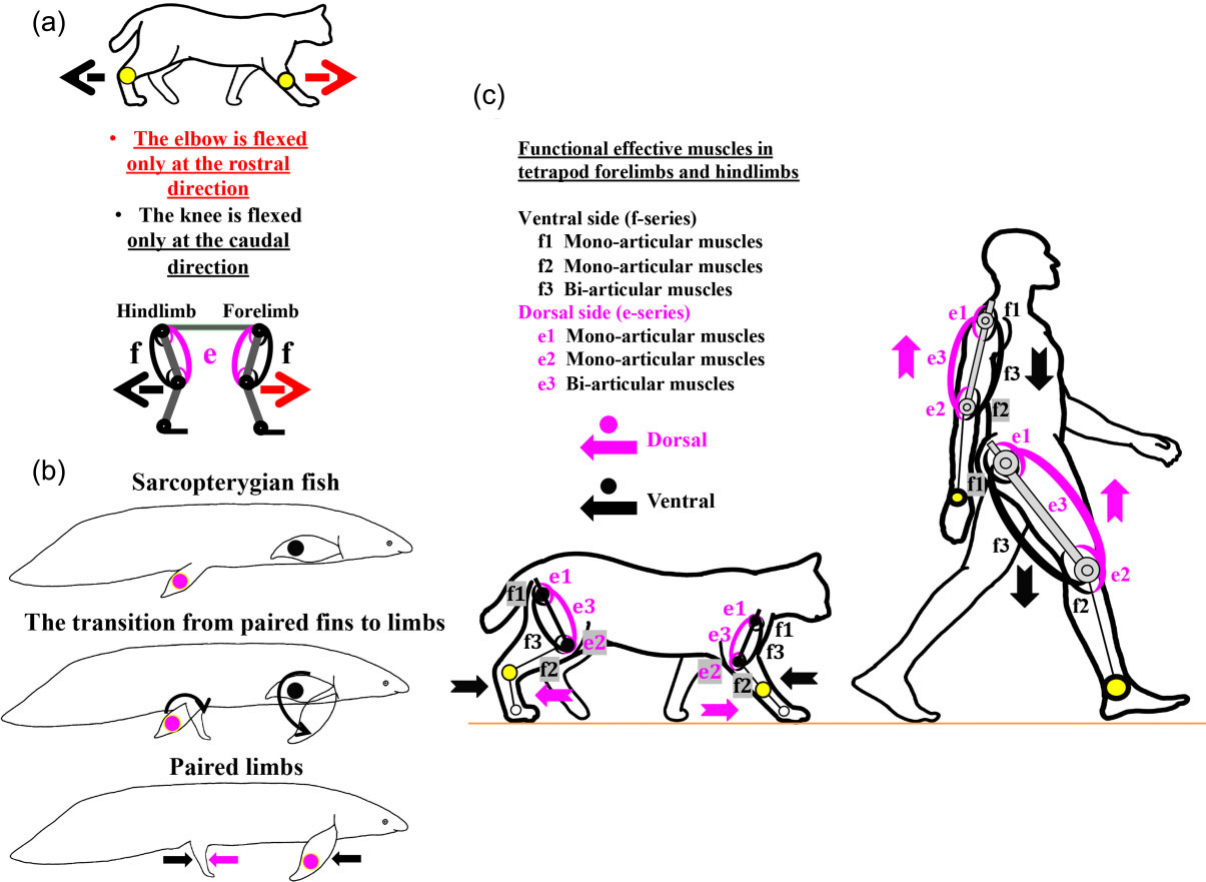
Biological prerequisites for an application of the two-joint link model to studies of tetrapod limb locomotion. (**A**) The configuration of tetrapod limbs and direction of flexion and extension. The elbow is flexed only in the rostral direction, whereas the knee is flexed only in the caudal direction. (**B**) Evolution of the orientations of paired fins in sarcopterygian fishes and paired limbs in tetrapods through the fin-to-limb transition. (**C**) Biological orientations of tetrapod and human forelimbs and hindlimbs and locations of e-series and f-series muscles in forelimb and hindlimb. The dorsal side and ventral side of limbs differ in forelimbs and hindlimbs due to the evolutionary history of tetrapod limbs through the fin-to-limb transition in Late Devonian and Early Carboniferous Periods (Romer 1971; Shubin et al. 2006; Johanson et al. 2007; Clack 2012; Miyake et al. 2016; Cloutier et al. 2020). The dorsal and ventral sides of human forelimbs and hindlimb match the map of dermatomes and cutaneous nerve territories (Schünke et al. 2017; Martini et al. 2018).

The two-joint link model clearly requires clarification of the orientation of mono- and bi-articular muscles in forelimbs and hindlimbs prior to the application of the model to studies of tetrapod locomotion. In all tetrapods, the knee is flexed only in the caudal direction, whereas the elbow is flexed only in the rostral direction (Fig. 6A). This is an important aspect of limb locomotion. As far as we know, there are no exceptions among tetrapods (Romer 1966 and 1971; Carroll 1990 and 2009). With this configuration of tetrapod forelimbs and hindlimbs, an arrangement of e-series and f-series mono- and bi-articular muscles in tetrapod limbs is proposed in our present review (Fig. 6), as the two-joint link model proposed that sequential switches of output forces occur counterclockwise or clockwise around the 360-degree perimeter of the wrist or the ankle, respectively, (Figs. 4 and 5).

To verify the arrangement of e-series and f-series muscles, we first need to define biological orientations of paired fins and paired limbs, as paired fins of sarcopterygian fishes have been proposed to have evolved into paired limbs of tetrapods during and/or after the fin-to-limb transition in the Late Devonian and Early Carboniferous (Shubin et al. 2006; Johanson et al. 2007; Clack 2012; Miyake et al. 2016; Cloutier et al. 2020). Romer (1971) stated that “upper and lower surfaces—back and belly aspects—are reasonably named dorsal and ventral” in vertebrates including humans (pages 7–9, Fig. 3). To summarize Romer's description (1971) of the evolution of tetrapod limbs from fish paired fins, Fig. 6B illustrates the orientation of paired fins as they evolved to become paired limbs. Pectoral fins of teleost and sarcopterygian fishes are normally positioned such that the ventral side faces laterally, whereas pelvic fins are normally positioned such that the dorsal side faces dorsally (Fig. 6B). During the fin-to-limb transition, pectoral fins rotated dorsoventrally as they evolved into forelimbs. Therefore, the first digits of forelimbs were turned toward the midline of the body. Pelvic fins stayed as they were and evolved into hindlimbs. However, pelvic fins rotated caudo-rostrally with evolutionary changes in the limb joints, and thus the dorsal sides of the fins face rostrally (Romer 1971). As Pierce et al. (2012) pointed out, these evolutionary changes enabled hindlimbs to perform a long-axis rotation and enabled the plantar surface to make contact with the ground (Fig. 6B).

With respect to the anatomical position of humans, a person adopts an upright posture with his or her forelimbs positioned straight downward with the palm facing forward, which represents the ventral side of the forelimbs. In contrast, a person with upright posture has the dorsal side of his or her hindlimbs facing straight forward (Schünke et al. 2017; Martini et al. 2018). These orientations of human forelimbs and hindlimbs match the map of their dermatomes and cutaneous nerve territories (Schünke et al. 2017; Martini et al. 2018). Therefore, the dorsal side and ventral side differ between the forelimbs and hindlimbs in humans and other tetrapods, and the arrangement of e-series and f-series muscles that the two-joint link model proposed thus becomes applicable as our biological prerequisites for studies of tetrapod locomotion. Fig. 6C illustrates the arrangement of e-series and f-series muscles in forelimb and hindlimb of a cat, as an example of non-human tetrapods and humans. On the dorsal side of both forelimbs and hindlimbs, e-series muscles are located, where f-series muscles are located on the ventral side of both the limbs. Thus, we propose (1) that sequential switches (counterclockwise in forelimbs and clockwise in hindlimbs) of output forces at three different axes occur as the reflection of the configuration of tetrapod forelimbs and hindlimbs (Fig. 6A) and (2) that locations of e-series and f-series muscles in forelimbs and hindlimbs (Fig. 6C) are biological characteristics of the two-joint link model.

## Contact task and postural control

Humans are able to stand, walk, jump, and/or run, with their posture control. If a person is standing upright, he or she should be able to control his or her balance without much sway or slippage. Unfortunately, we typically do not think about this issue, and we do not even realize that we need to overcome and/or withstand gravity to make all terrestrial locomotive activities possible. When engineers wish to make a robot that is able to stand, walk, jump, and/or run without difficulties, however, this issue becomes quite problematic and critical. As defined in the field of robotic engineering, contact tasks are regarded as a set of interactions that involve friction. Thus, once a robotic leg touches the ground, the robot needs to counteract the force from the ground (ground reaction forces) with the friction between the robotic leg and the ground while controlling its posture without slippage. Unless the robot is able to control a contact task and its posture, it will not be able to stand and move at all. In addition, most robots have been built with a motor (actuator) in each joint, and highly sophisticated mathematical and engineering computational modeling has been needed to control all joints in concert to achieve contact tasks and postural control (Vukobratovic et al. 2003; Vukobratovic et al. 2009).

**Fig. 7:**
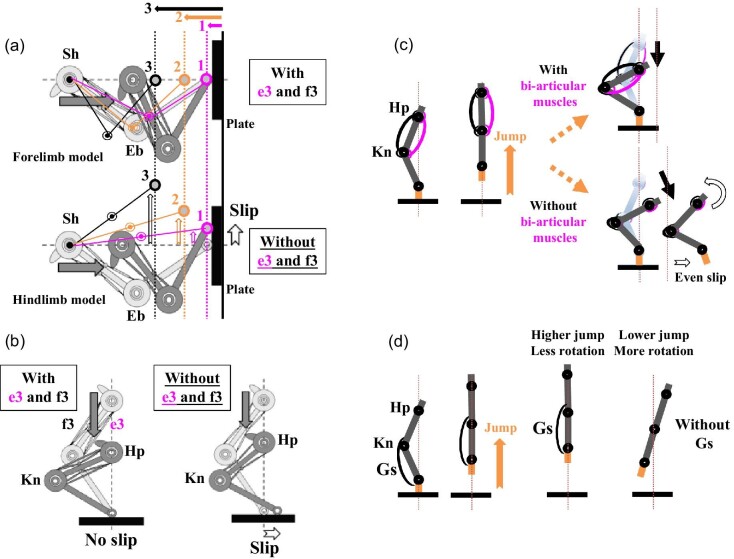
A series of contact tasks with human mono- and bi-articular muscles and the gastrocnemius muscle in the two-joint link model. (**A**) A mechanical test of the two-joint link model with or without a pair of bi-articular muscles (e3 and f3) in the forelimb (Fujikawa et al. 2001). As the plate was moved toward the model from position 1 to position 3, the link model was pushed against the plate. The researchers tested whether the model was able to make a correct trajectory and stay on the plate or slipped with or without the bi-articular muscles. (**B**) A mechanical test of the two-joint link model with or without a pair of bi-articular muscles (e3 and f3) in the hindlimb (Oshima et al. 2010; Oshima et al. 2017). (**C**) Illustrated jumping motions that show the requirement of a pair of bi-articular muscles for landing without any slipping or rotation (Oshima et al. 2004; Oshima et al. 2017). (**D**) A role for the gastrocnemius muscle of the hindlimb during higher jumping motions to prevent much rotation of the limb (Kanayama et al. 2001; Oshima et al. 2005). Eb: elbow joint; Gs: gastrocnemius muscle; Hp: hip joint; Kn: knee joint; Sh: shoulder joint.

Given these difficulties, it is noteworthy that the two-joint link model of mono- and bi-articular muscles in the human brachium and thigh provided a part of solutions for contact tasks without the need for sophisticated mathematical and engineering control of joints simultaneously. Fujikawa et al. (2001) examined whether a robotic arm model equipped with mono- and bi-articular muscles would be able to control directional movements (trajectory movements) of the arm and carry out a contact task when the model would push the plate at the end with or without a pair of bi-articular muscles (Fig. 7A). With a pair of bi-articular muscles, the model achieved accurate trajectory movements, whereas the movements were off line in their absence (Fujikawa et al. 2001). In addition, as illustrated in Fig. 7A, when the plate moved from position 1 to 3, the model without a pair of bi-articular muscles slipped on the plate each time when it was pushed against the plate and thus could not control the contact task (Fig. 7A). This study demonstrated clearly that the model without a pair of bi-articular muscles was not able to counteract the force from the plate (ground reaction forces) with the friction between the model and the plate. In addition, Fujikawa and Oshima (2010) and Oshima et al. (2017) analyzed whether a mechanical leg model was able to stand upright and control its posture without much slippage with or without a pair of bi-articular muscles. Again, without a pair of bi-articular muscles (Fig. 7B), the model slipped and was even rotated in some cases. Therefore, a pair of bi-articular muscles along with mono-articular muscles in the brachium and thigh play a role in contact task achievement against the ground reaction force and/or in postural control (Fujikawa 2016).

In addition, Kanayama et al. (2001), Oshima et al. (2004), and Oshima et al. (2005) have tested the role of bi-articular muscles and the gastrocnemius in contact tasks and postural control during a jumping motion using the robotic leg model (Fig. 7C, D). According to their studies, a pair of bi-articular muscles in the model did achieve the contact task without slippage and/or rotation (Fig. 7C). In addition, the gastrocnemius prevented the model from being rotated when the model touched the ground (Fig. 7D). However, without a pair of bi-articular muscles, the gastrocnemius alone was not able to control this contact task. In addition, Nakagawa et al. (2015) showed that the rectus femoris (e3), vastus medialis (e2), and gastrocnemius were all activated when a human subject landed on the floor from a vertical jump and suggested that these muscles were able to counteract the ground reaction force and control the contact task. Oshima et al. (2004) concluded that “the line of action invariably passed near the center of gravity and the conversion of elastic energy to rotational energy was suppressed.” This action was proposed to be the torque transfer function by which output forces of mon- and bi-articular muscles would be passed on to the distal joints including the ankle (Oshima et al. 2004; Oshima et al. 2010). In fact, the torque transfer function has been discussed as the transfer of mechanical energy of bi-articular muscles between different joints, in particular between proximal joints and distal joints, during locomotive activities (Cleland 1867; Elftman 1939; Prilutsky and Zatsiorsky 1994; Biewener and Daley 2007; Carroll and Biewener 2009). Altogether, the pair of bi-articular muscles in the brachium and thigh has been proposed to play a role in contact task achievement and/or postural control against the ground and/or objects in a normal gravity environment.

The two-joint link model of antagonistic pairs of mono- and bi-articular muscles in the brachium and thigh was, therefore, established based on biomechanical analyses of human subjects and theoretical and mechanical testing using robotic limbs (Fujikawa 2016). The model proposes the followings: (1) Mono- and bi-articular muscles coordinately exert their output forces and directions at the wrist or the ankle, neither at the elbow nor at the knee (Figs. 4 and 5). The relative strength of a different combination of mono- and bi-articular muscles exerts a combined force and determines directional movement of the entire forelimb or hindlimb between two of the three axes; (2) Sequential switches in the output force occur in a counterclockwise and clockwise fashion around the 360-degree perimeter of the wrist and the ankle, respectively, (Figs. 5 and 6); (3) Antagonistic pairs of mono- and bi-articular muscles play a role in directional movements, contact tasks, and/or postural control (Fig. 7); (4) An antagonistic pair of bi-articular muscles in the brachium and thigh engages the torque transfer function from proximal joints to distal joints.

## Applications of the two-joint link model for gait analyses

The two-joint link model has been modeled and tested based on biomechanical EMG analysis, mathematical and computational modeling and mechanical engineering robotic models of the forelimb and hindlimb. The advantage of this approach is its theoretical and mechanical modeling power to test underlying experimental assumptions and hypotheses (Gravish and Lauder 2018). Its drawback, however, is the difficulty of directly applying such mechanical engineering modeling to experiments in the field of biology. Therefore, we wish to discuss several possible applications of the two-joint link model to studies of limb locomotion including gait analyses by applying two theoretical and mathematical premises of the model in addition to the prerequisites that we have proposed above.

**Fig. 8:**
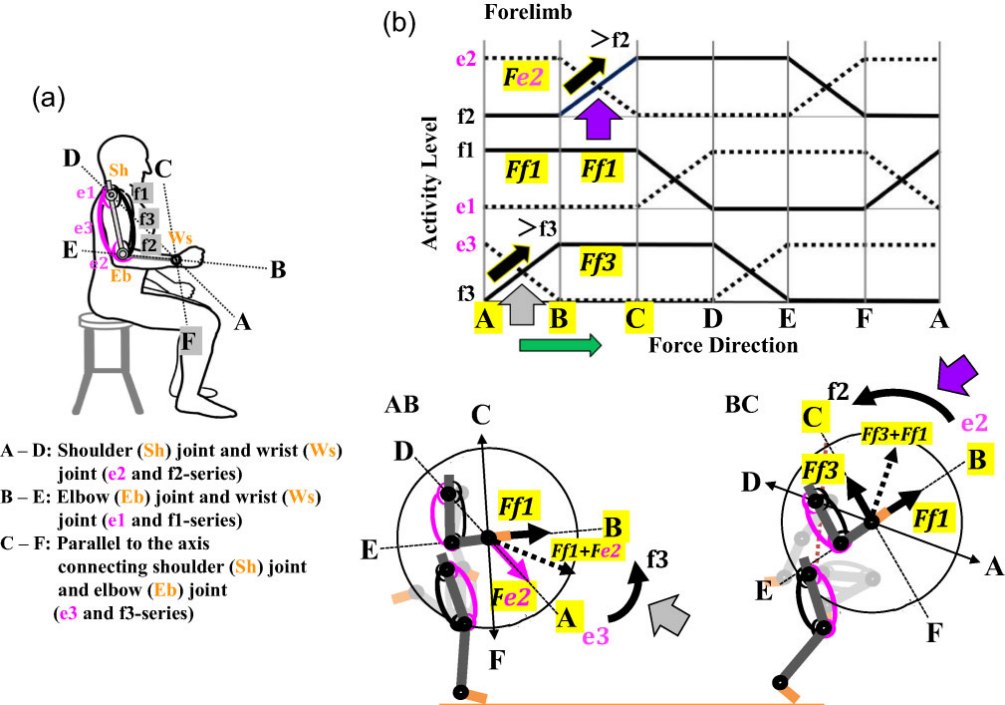
Predicted output forces in the human forelimb during a sprint performance in light of the two-joint link model. (**A**) Defined axes: A–D (e2 and f2 series muscles); B–E (e1 and f1 series muscles); C–F (e3 and f3 series muscles). (**B**) Two examples of counterclockwise switches in activity, e3 to f3 (AB) and e2 to f2 (BC), and output combined forces, *Fe2* + *Ff1* and *Ff1* + *Ff3*, respectively, with the schematized IEMGs, when the human right forelimb moves in coordination with the contralateral hindlimb during a sprint performance. A magnitude of each combined force will determine the force direction and thus the directional movement of the forelimb. Large arrows: switches of output forces in AB and BC.

**Fig. 9:**
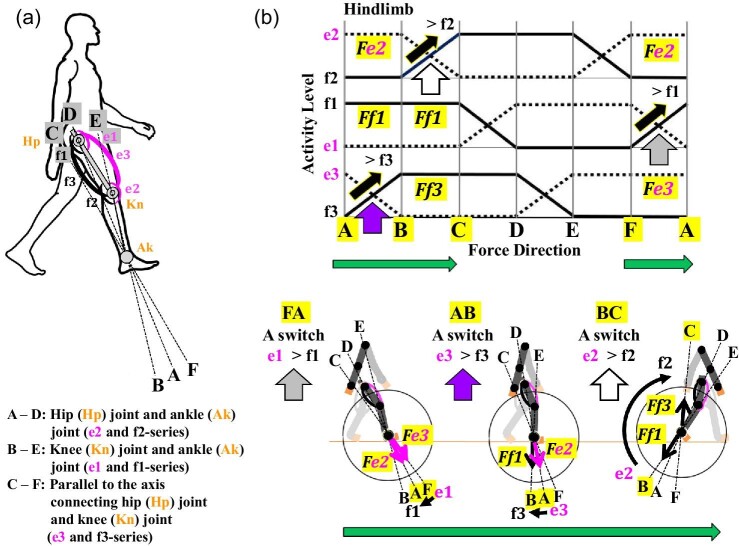
Predicted output forces in the human hindlimb during the stance phase of the bi-pedal walking gait in light of the two-joint link model. (**A**) Defined axes: A–D (e2 and f2 series muscles); B–E (e1 and f1 series muscles); C–F (e3 and f3 series muscles). (**B**) Three stages of the stance phase and concomitant clockwise switches in activity, e1 to f1 (FA), e3 to f3 (AB), and e2 to f2 (BC) with changes in the combined output forces, *Fe3* + *Fe2*, *Fe2* + *Ff1*, and *Ff1* + *Fe3*, respectively, as depicted in the schematized IEMGs. The magnitude of a combined force will determine the force direction and thus the direction of hindlimb. Large arrows: switches of output forces in FA, AB, and BC.

The first premise of the two-joint link model that is relevant and essential here is an establishment of theoretical and experimental axes among the three joints of the brachium or thigh: A–D is the axis between the shoulder joint and the wrist joint or the axis between the hip joint and the ankle joint, B–E is the axis between the elbow joint and the wrist joint or the axis between the knee joint and the ankle joint, and C–F is the axis parallel to the axis connecting the shoulder joint and the elbow joint and to the axis connecting the hip joint and the knee joint (Figs. 8A and 9A). The ranges AB and DE represent the shoulder-wrist angle in forelimbs and the hip-ankle angle in hindlimbs, BC and EF represent the elbow angle in forelimbs and the knee angle in hindlimbs and CD and FA represent the shoulder-humerus angle in forelimbs and the hip-femur angle in hindlimbs. These three axes represent the direction of the output forces exerted by e2 and f2 (A–D), e1 and f1 (B–E), and e3 and f3 (C–F). Therefore, two of the six axes limit an angle, for instance, BC by B–E and C–F, but the actual magnitude of each angle will vary with a type of tetrapod animals, different individuals, their different postures and/or different activities of limb locomotion (Figs. 8A and 9A).

The angles of the right forelimb in human sprint gait (Fig. 8B) and those of the right hindlimb in human bi-pedal walking gait (Fig. 9B) illustrate how they would vary. For the right forelimb in the sprint gait, a combined force *Ff1 *+ *Ff3* is exerted in BC where the elbow becomes flexed, since the elbow angle (BC) changes due to the action of f3 as a sprinter runs after the switch AB changes to BC (Fig. 8B). For the right hindlimb in the bi-pedal walking gait, a combine force *Ff1 *+ *Ff3* is exerted in BC where the knee becomes extended, since the knee angle (BC) changes due to the action of f3 as a person is walking after the switch FA changes to AB to BC (Fig. 9B). Note that the elbow and the knee move at opposite directions, counterclockwise and clockwise, respectively, even though the same combined force *Ff1 *+ *Ff3* is exerted (Figs. 8B and 9B).

As illustrated above, the second premise is, therefore, that there is a set of output force directions: counterclockwise in the forelimb and clockwise in the hindlimb (Figs. 4 and 5). This directional difference represents evolutionary changes and comparative anatomical differences in the tetrapod forelimb and hindlimb (Fig. 6A, B). As the biological prerequisite, e-series and f-series muscles are located each on the dorsal side and the ventral side of both forelimbs and hindlimbs, respectively, (Fig. 6C).

### Forelimb locomotion in the sprint performance of humans

Unlike human bi pedal walking gait, running or sprinting extensively uses both forelimbs and hindlimbs. An individual right forelimb is fully flexed as the contralateral hindlimb becomes flexed, the stride that approaches the recovery stage of sprint running (Mero et al. 2015). Although the role of forelimbs in running or sprinting remains unclear (Harrison 2010; Macadam et al. 2018; Bezodis et al. 2019), their movements are clear to all of us when we engage in such activities. We thus show two illustrated figures of sprint performance based on a video of a female sprinter on a treadmill (Clark et al. 2017). By applying the two premises of the two-joint link model, output forces and directions are proposed at three axes during the braking stage of the sprint performance (Haugen et al. 2019; Fig. 8B). During this stage, as the sprinter moves forward, her right forelimb moves as its flexion in a counterclockwise direction. Depending on the relative strength of the output forces, *Ff1* and *Fe2* or *Ff1* and *Ff3*, the output force directions can be calculated as a combined force, *Ff1* + *Fe2* or *Ff3* + *Ff1*, in the right forelimb. In AB, the bi-articular muscles e3 and f3 are predicted to switch their activities, whereas f1 (deltoid anterior) and e2 (triceps brachii lateral head) are predicted to exert a combined force (*Ff1* + *Fe2*) and determine the direction of the forelimb movement (Oshima 2008). Sequentially in BC, the mono-articular muscles e2 and f2 are first predicted to switch their activities (Fig. 8B), whereas f1 (deltoid anterior) and f3 (biceps brachii long head) are then predicted to exert a combined force (*Ff3* + *Ff1*) and determine the direction of the forelimb movement (Oshima 2008). The right forelimb would thus make a flexing swing when a sprinter tries to accelerate toward the flight stage of his or her sprint (Wiemann and Tidow 1995; Morin et al. 2015; Macadam et al. 2018). As described below, the contralateral left hindlimb would make a flexing swing as a part of diagonal couplet lateral sequence gait.

### Hindlimb locomotion in human bi-pedal walking gait

We can also apply the two premises of the two-joint link model to human bi-pedal walking gait (Fig. 9). Fujikawa et al. (2010) examined human bi-pedal walking gait in light of the two-joint link model and reported some of the data that we present here as an example of the application of the two-joint link model to such an

analysis. The bi-pedal walking gait in humans consists of the stance and swing phases, during which the left and right hindlimbs alternate their steps (Nymark et al. 2005; Sasaki et al. 2009; Supuk et al. 2014; Abu-Farj et al. 2015). At the beginning of the stance phase, one hindlimb, for instance, the right hindlimb, steps forward as its heel strikes the ground. Then, as the body moves forward, the right hindlimb supports the entire body weight until the heel of the left hindlimb steps forward and its heel touches the ground. To illustrate this event, we can consider three sets of figures with three axes and output forces and switches involving different antagonistic pairs of mono- or bi-articular muscles (Fig. 9B). During the sequence of the stance phase, the output forces and directions switch from FA to AB to BC, as an antagonistic pair of mono-articular muscles (e1 to f1 and e3 to f3) and bi-articular muscles (e2 to f2) each switch their activities. The relative strength of each output force (*Fe3* + *Fe2*, *Fe2* + *Ff1*, or *Ff1* + *Ff3*) determines their combined force and direction (Fig. 9A, B).

**Fig. 10:**
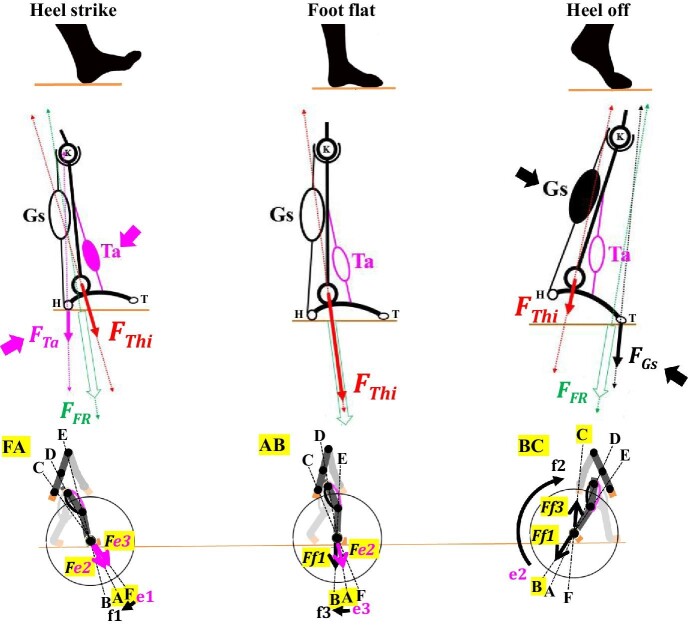
The predicted sequence of contact tasks during the stance phase of the human bi-pedal walking gait in light of the two-joint link model. During the stance phase, the foot first touches the ground with the heel (heel strike) and ultimately leaves the ground with the heel rising first, followed by the toes (heel off); at each step of this process the two-joint link model predicts sequential switches in the output force from FA to AB to BC (also see Fig. 9). The tibialis anterior (Ta) is activated when the heel is off the ground, whereas the gastrocnemius (Gs) is activated when the toes are off the ground. H: heel; K: knee; T: toe. *F_FR_*: a combined force by *F_Thi_* and either *F_Ta_* or *F_Gs_*; *F_Gs_*: output force from the gastrocnemius; *F_Ta_*: output force from the tibialis anterior; *F_Thi_*: output force from the mono- and bi-articular muscles in the thigh.

During the stance phase, each human hindlimb encounters ground reaction forces and needs to counteract these to maintain an individual's body balance and continue the bi-pedal walking gait cycle without any slippage and/or rotation (Marasovic et al. 2009). This represents the achievement of the contact task (Fig. 7). As described in Fig. 9, the two-joint link model predicts sequential switches in the output forces during the stance phase of the bi-pedal walking gait as a different combination of mono- and bi-articular muscles becomes active (Aizawa et al. 2008). Fig. 10 illustrates our proposed contact task during the stance phase of the bi-pedal walking gait in light of the two-joint link model. To achieve the contact task during the stance phase, the output force (*F_Thi_*) exerted at the ankle by a different combination of mono- and bi-articular muscles of the thigh and either the output force (*F_Ta_*) of the tibialis anterior (Ta) or the output force (*F_Gs_*) of the gastrocnemius will counteract as a geometrically combined force (*F_FR_*) against the ground reaction forces at the point of heel strike or heel off, respectively, (Fig. 10). Abe et al. (2014) demonstrated that the rectus femoris (e3), vastus medialis (e2), soleus, and tibialis anterior were all activated as an individual stands up based on an EMG analysis and these data have been confirmed with a robotic leg model (Abe et al. 2014). As illustrated in Fig. 10, the activities of these muscles are identical to these of the heel strike stage at the beginning of the stance phase during which *Fe3* + *Fe2* (*F_Thi_*) along with the force (*F_Ta_*) of the tibialis anterior exert together their combined output force (*F_FR_*) and direction. At the heel off stage, *Ff3 *+ *Ff1* (*F_Thi_*) along with the force (*F_Gs_*) exert together their combined force (*F_FR_*) and direction. The output forces (*F_Thi_*) exerted by mono- and bi-articular muscles in the thigh along with either the tibialis anterior or gastrocnemius at the ankle could be thus attributed to the torque transfer function of bi-articular muscles from the proximal to distal joints of the link model that Oshima et al. (2004) and Oshima et al. (2010) analyzed mathematically and mechanically.

There is one interesting study that has suggested a role of the bi-articular muscles in maintaining body balance. Using a novel device, an angular momentum perturbator (AMP), that perturbs a human subject carrying this device on her or his back, Schumacher et al. (2019) made EMG recordings from the muscles in the thigh and collected kinetic and kinematic data when the AMP was used to introduce perturbations in the forward and backward pitch of the upper body. The muscles examined were the vastus lateralis (e2), rectus femoris (e3), tensor fasciae latae and gluteus maximus (f1), biceps femoris long head (f3), tibialis anterior, gastrocnemius, and soleus. In response to forward pitch perturbations those muscles with the highest activity were the biceps femoris long head (f3), gastrocnemius, and soleus, whereas those with the highest activity in response to backward pitch perturbations were the rectus femoris (e3), vastus lateralis (e2), and tibialis anterior. They concluded that the bi-articular muscles with the greatest responses would have the most important role(s) in restoring upper body balance (Schumacher et al. 2019). Interestingly, the backward pitch perturbations are identical to the heel strike stage of the bi-pedal walking gate in which e3 and e2 are predicted to exert their combined output force along with the force (*F_Ta_*) exerted by the tibialis anterior. Similarly, the forward pitch perturbations are identical to the heel off stage of the waking gate in which f3 and f1 are predicted to exert a combined output force along with the force (*F_Gs_*) exerted by the gastrocnemius (Fig. 10).

The muscles of the lower leg, in particular, the tibialis anterior and gastrocnemius, have important roles in adjusting body balance, although these muscles do not constitute a part of the thigh (Di Giulio et al. 2009; Day et al. 2013). Although a number of studies have focused on a single muscle or a group of muscles in the lower leg, including the tibialis anterior, gastrocnemius, soleus, and/or the muscles associated with the ankle joint and pedis (Masani et al. 2003; Di Giulio et al. 2009; Zehr et al. 2009; Day et al. 2013; Boonstra et al. 2015; Valle et al. 2016; Usherwood and Granatosky 2020), the antagonistic pairs of mono- and bi-articular muscles in the thigh have rarely been regarded as a contributor to contact task. An antagonistic pair of bi-articular muscles alone were proposed to have a role in contact task (Fujikawa et al. 2001; Kanayama et al. 2001; Oshima et al. 2004; Oshima et al. 2005; Fujikawa and Oshima 2010; Oshima et al. 2017). In addition, all possible locomotive activities of limbs including standing, walking, running, or sprinting, and/or other activities require the achievement of contact task (Biewener and Daley 2007; McKay et al. 2007; Marasovic et al. 2009; Weyand et al. 2010; Clark et al. 2017; Pavei et al. 2019).

## The diagonal-couplet lateral sequence of tetrapods

**Fig. 11:**
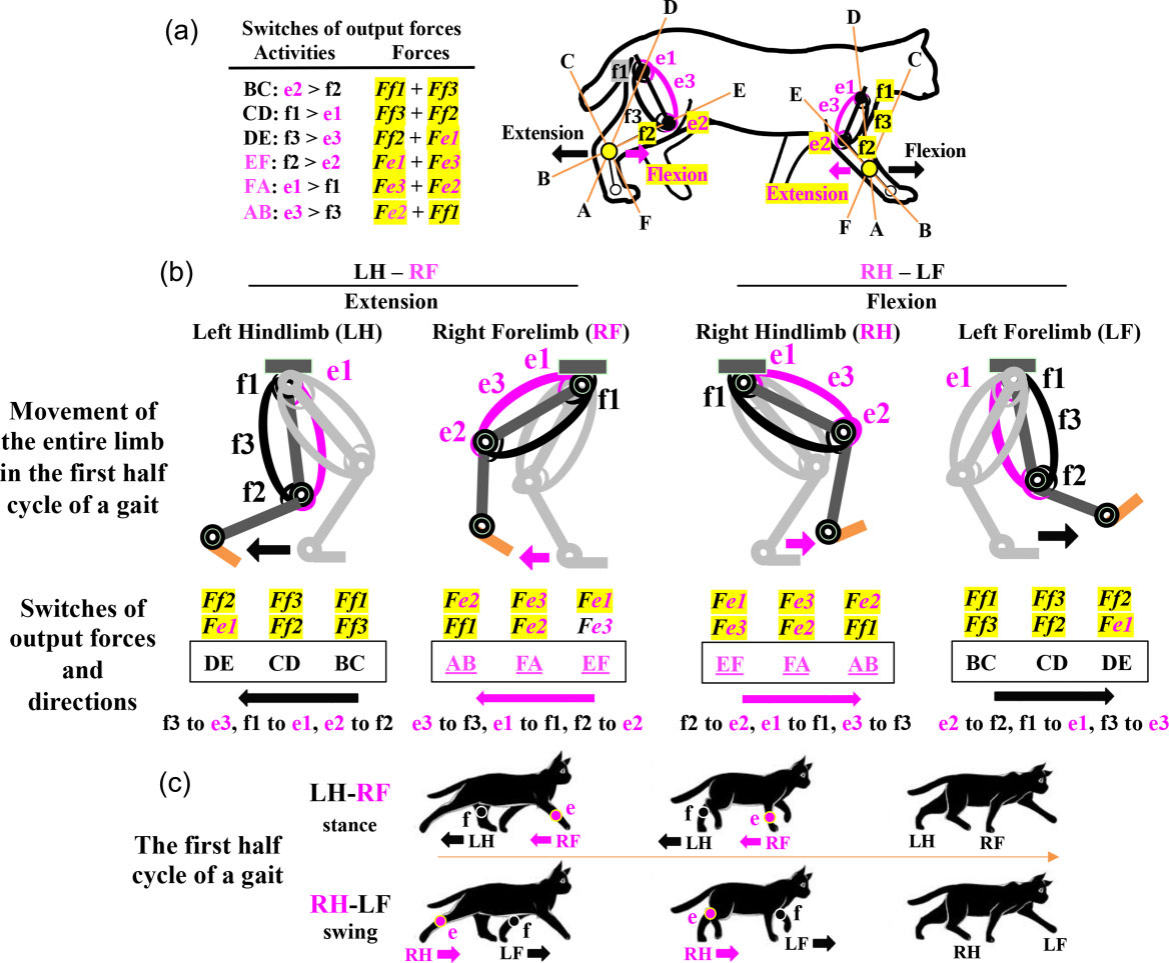
Proposed activities of mono- and bi-articular muscles in a diagonal-couplet lateral sequence based on the two-joint link model. (**A**) The switches in output forces and the two-joint link model with three axes and the distribution of e-series and f-series muscles in tetrapod forelimbs and hindlimbs. (**B**) Switches in output forces during the extension and flexion of two pairs of couplets, the left hindlimb and right forelimb (LH–RF) and the right hindlimb and left forelimb (RH–LF), respectively, in the first half cycle of a gait. At the stance phase, the extension of LH–RF goes through BC to CD to DE (left hindlimb, LH) and EF to FA to AB (right forelimb, RF). At the swing phase, the flexion of RH–LF goes through EF to FA to AB (right hindlimb, RH) and BC to CD to DE (left forelimb, LF). (**C**) The proposed sequence of the LH–RF couplet and RH–LF couplet during the first half cycle of a diagonal-couplet lateral sequence. The LH–RF couplet makes the extension of LH and RF as the stance phase, although LH and RF go through different switches, BC to CD to DE (f: f-series muscles) and EF to FA to AB (e: e-series muscles), respectively. RH–LF couplet makes the flexion of RH and LF as the swing phase, although RH and LF go through different switches, EF to FA to AB (e: e-series muscles) and BC to CD to DE (f: f-series muscles), respectively. LF: left forelimb; LH: left hindlimb; RF: right forelimb; RH: right hindlimb.

Based on the two-joint link model and our biological prerequisites we propose (Figs. 5 and 6), the activities of mono- and bi-articular muscles in the quadrupedal gait will be proposed as a diagonal-couplet lateral sequence (Fig. 11; Bininda-Emonds et al. 2007; Nyakatura et al. 2014; Aydin et al. 2017). The e-series muscles are located on the dorsal side of forelimbs and hindlimbs, whereas the f-series muscles are located on the ventral side of both the limbs (Figs. 6C and 11A). Suppose that a tetrapod starts a quadrupedal walking with the left hindlimb (LH) and right forelimb (RF) (LH–RF couplet as a diagonal-couplet) and with the right hindlimb (RH) and the left forelimb (LF) (RH–LF couplet as a diagonal-couplet) in the first half cycle of a gait (based on cats, personal observations) (Fig. 11B, C). During the extension of the LH–RF couplet as the stance phase, output forces *Ff1* + *Ff3* to *Ff3* + *Ff2* to *Ff2* + *Fe1* are generated in LH with sequential switches of a different antagonistic pair of mono- or bi-articular muscles (e2 to f2, f1 to f2, f3 to e3) in BC to CD to DE, whereas output forces *Fe1* + *Fe3* to *Fe3* + *Fe2* to *Fe2* + *Ff1* are generated in RF with sequential switches of a different antagonistic pair of mono- or bi-articular muscles (f2 to e2, e1 to f1, e3 to f3) in EF to FA to AB. During the flexion of RH–LF couplet as the swing phase, output forces *Fe1* + *Fe3* to *Fe3* + *Fe2* to *Fe2* + *Ff1* are generated in RH with sequential switches of a different antagonistic pair of mono- or bi-articular muscles (f2 to e2, e1 to f1, e3 to f3) in EF to FA to AB, whereas output forces *Ff1* + *Ff3* to *Ff3* + *Ff2* to *Ff2* + *Fe1* are generated in LF with sequential switches of a different antagonistic pair of mono- or bi-articular muscles (e2 to f2, f1 to f2, f3 to e3) in BC to CD to DE (Fig. 11B).

Therefore, in the quadrupedal gait as the diagonal-couplet lateral sequence, we propose the following sequences (Fig. 11C). In the first half of the gait, the LH–RF couplet proceeds as the stance phase with the sequential activity of the f-series (f, LH) muscles (extension) in BC to CD to DE and the e-series (e, RF) muscles (extension) in EF to FA to AB. Similarly, the RH–LF couplet proceeds as the swing phase with the sequential activity of the e-series (e, RH) muscles (flexion) in EF to FA to AB and the f-series (f, LF) muscles (flexion) in BC to CD to DE (Fig. 11B, C). Although the last half of the gait is not shown here, the LH–RF and RH–LF couplets each go through two sequences of switches, EF to FA to AB (LH) and BC to CD to DE (RF) or BC to CD to DE (RH) and EF to FA to AB (LF), respectively, during the swing phase and stance phase, respectively.

As illustrated in Fig. 8B, a sprint performance involves extensive movements of not only hindlimb but also forelimbs (Macadam et al. 2018). For instance, when a sprinter enters the stage just prior to his flight phase, the right forelimb (RF) is fully flexed as the contralateral left hindlimb (LH) becomes flexed, whereas the left forelimb (LF) is fully extended as the contralateral right hindlimb (RH) become extended (Mero et al. 2015). As the interlimb and intersegmental coordination (Grillner 1981; Zehr et al. 2009; Sürmeli et al. 2011; Karakasiliotis et al. 2016; Côté et al. 2018), these movements of two contralateral pairs of forelimbs and hindlimbs are identical to the diagonal-couplet lateral sequence of quadrupedal tetrapods (Fig. 11C; Courtine et al. 2005; Choi and Bastian 2007; Ogihara et al. 2010; Higurashi et al. 2019; Grillner and Manira 2020). Our proposed model of the diagonal-couplet lateral sequence will hopefully thus be tested against a large number of quadrupedal and bi-pedal gait studies in a variety of tetrapods including humans ([Bibr bib137][Bibr bib137]; Bates and Schachner 2012; Allen et al. 2014; Hutchinson et al. 2015; Karakasiliotis et al. 2016; Daley and Birn-Jefferty 2018), since these studies have contributed enormously to our understanding of tetrapod locomotion and robotic engineering (Russel and Bels 2001; Ijspeert et al. 2005; Biswal and Mohanty 2021).

The parameters in the two-joint link model have been set up and verified based on controlled experiments, in particular, evenly distributed ranges from AB to FA around the perimeter of the wrist joint or the ankle joint and the maximum output forces applied. However, as shown in examples of the application of the model (Fig. 7, Fig. 8, Fig. 9), the angles bound by two of three axes, AD, BE, CF, would change considerably, depending on limb movements, different postures or strides, and/or and different activities of limb locomotion. Any output forces exerted by muscles would also vary considerably across different phases of limb movements. The combined forces and directions in six ranges need to be measured and determined for motion analyses. Since motion capture system, a force plate, force sensors, muscle activities, three-dimensional analyses, and/or computerized analyses all become powerful tools and available for locomotion studies (Oshima 2008; Karakasiliotis et al. 2016; Fischer et al. 2018; Seth et al. 2018; Manafzadeh et al. 2021; Gatesy et al. 2022), real-time observations of the motion of a subject and joint movements, EMG recordings, recordings of output forces, and recordings of ground reaction forces will facilitate an application and advancement of the two-joint link model for studies of locomotion in humans and other tetrapods.

## Issues of the two-joint link model that remain to be clarified

As the two-link joint model has been established based on human limb locomotion and has been tested using mechanical engineering robotic models, there will be some issues to be discussed and/or clarified for its application with respect to studies of tetrapod locomotion. Here, we present two issues as examples.

### A sudden change in orientation and/or irregular behavioral movements

The original studies of the two-joint link mechanism were performed with different horizontal and vertical directions for the defined movements of forelimb or hindlimb (Fig. 3; Fujikawa et al. 1996; Fujikawa et al. 1997; Oshima et al. 1999; Oshima 2008). Although all changes produced the predicted patterns with respect to output forces and directions, these patterns were generated when the experiment was set up such that the human subject underwent one set of defined movements at one set of fixed angles (without changes to the remaining angles) and then continued to a subsequent experimental setup with another set of defined movements at another set of fixed angles. Fujikawa et al. (1997) carried out the experiments by setting up human subjects to make isometric pull and push movements of the entire forelimb in the transverse plane (Fig. 3B). Even, they changed θ_1_ (the angle of shoulder flexion) but did change θ_ 2_ (the external angle of elbow flexion), 80^0^ and 80^0^,37^0^ and 120^0^,50^0^ and 90^0^,60^0^ and 60^0^ , and 42^0^ and 57^0^, respectively, in order for human subjects to make the isometric movements to a narrow range of the plane in front of human subjects (Fig. 5; Fujikawa et al. 1997). Unfortunately, to observe EMG activities of participating muscles for a sudden change in orientation and/or irregular behavioral movements, these coordinated changes of θ_1_ and θ_2_ did not allow more variable movements of the forelimb at all.

Therefore, the original experiments of the two-joint link model did not involve any sudden and/or irregular changes due to behavioral changes or due to the presence of an obstacle and/or uneven ground conditions in any one of the three angles. Furthermore, e-series and f-series muscles of the two-joint link model have been defined without treating a part of a given muscle or a muscle group that acts depending on the limb position individually (Oshima 2008). For instance, as the human deltoid muscle is composed of the anterior (clavicular), intermediate (acromial), and posterior (spinal) parts, it is generally regarded as the abductor of forelimbs (Schünke et al. 2017; Martini et al. 2018). In fact, the intermediate part serves as the abductor of forelimbs. However, the anterior part acts as a f1-series muscle to flex, rotate medially or adduct forelimbs whereas the posterior part acts as an e1-series muscle to extend, rotate laterally or adduct forelimbs (Oshima 2008). Depending on specific directional movement of forelimbs, a different part of the deltoid muscle will be thus activated individually or coordinately with other parts for a specific directional movement of forelimbs (Bernasconi et al. 2006; Hedt et al. 2020).

**Fig. 12:**
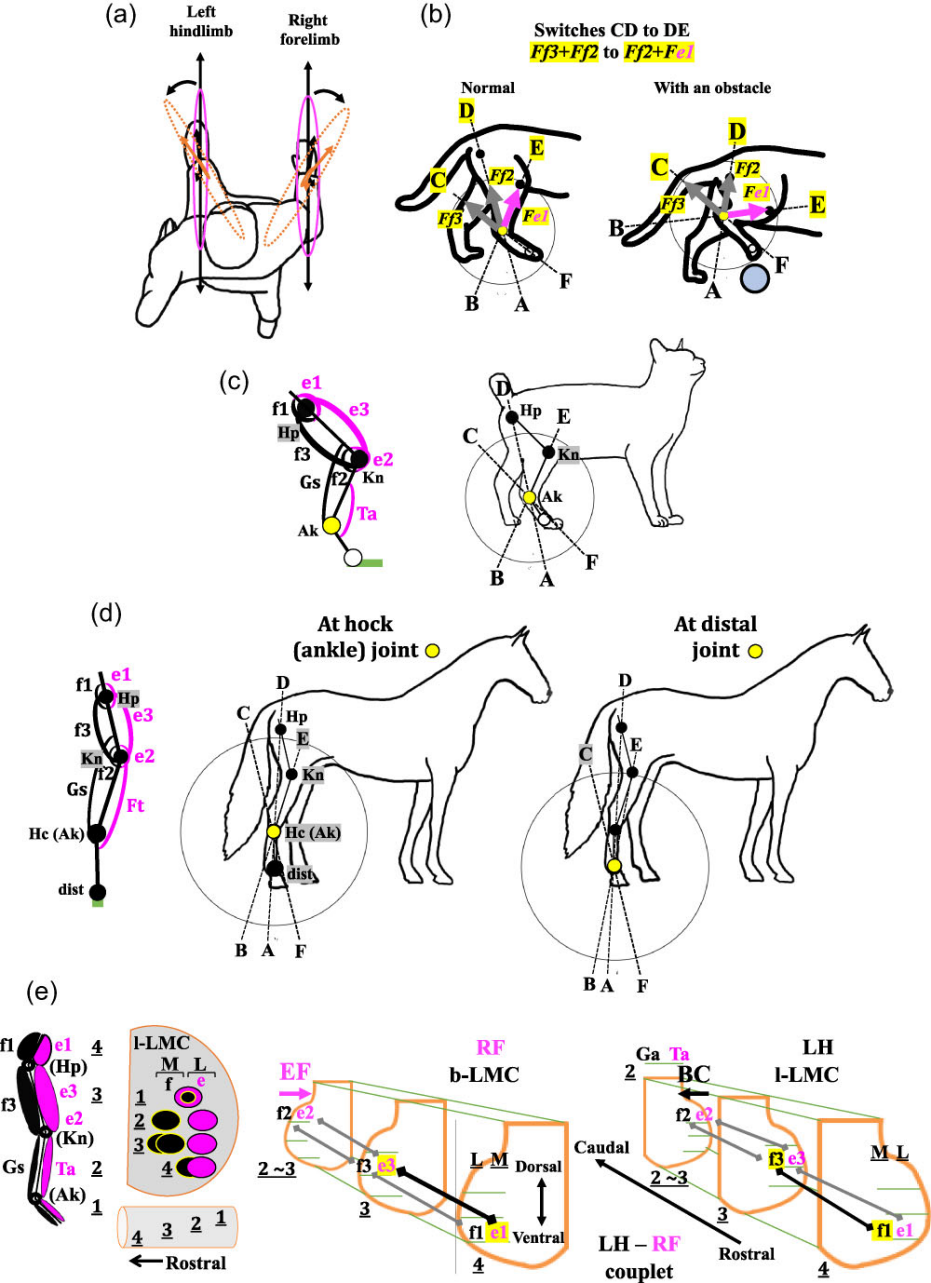
Four examples of unanswered questions related to the two-joint link model of mono- and bi-articular muscles in human forelimbs and hindlimbs and a predicted arrangement of motor pools for mono- and bi-articular muscles in the LMCs. (**A**) A sudden change in the direction of movement of a forelimb or a hindlimb during normal quadrupedal walking. (**B**) Predicted switched (CD to DE) and output forces in normal quadrupedal walking gait and an obstacle avoidance gait in cats based on EMG data and the gait analyses (Widajewicz et al. 1994; McFadyne et al. 1999; McKay et al. 2007; Markin et al. 2012; Frigon et al. 2015; Chu et al. 2017). (**C**) The two-joint link model in hindlimb of a digitigrade cat. (**D**) The two-joint link model in hindlimb of an unguligrade horses with another set of bi-articular muscles between the knee joint and the hock (ankle) joint. There might be two possible points where the e-series and f-series muscles in the thigh exert their forces at the hock (ankle) joint or at a more distal joint such as the fetlock joint, pastern joint, or coffin joint. (**E**) Motor pools in the branchial lateral motor column (b-LMC) and the lumber lateral motor column (l-LMC). The arrangement of e-series and f-series muscles in a hindlimb corresponds topologically to the arrangement of the motor pools in the lumber lateral motor column (l-LMC) that innervate these muscles (VanderHorst and Holstege 1997; Sürmeli et al. 2011; Gross et al. 2017). Potential activation sequences of motor neurons for mono- and bi-articular muscles are predicted if a tetrapod starts quadrupedal walking with the left hindlimb (LH) and right forelimb (RF) (LH–RF couplet) in the first half cycle of a gait based on our proposed activities of mono- and bi-articular muscles in a diagonal-couplet lateral sequence (Fig. 11C). Ak: ankle joint; dist: distal joint(s); Ft: fibularis tertius; Gs: gastrocnemius; Hc: hock joint; Hp: hip joint; Kn: knee joint; L: the lateral side of LMCs; M: the medial side of LMCs; Ta: tibialis anterior.

The question is thus what would happen to output forces and directions if a sudden change in the orientation of the forelimb or hindlimb is made due to behavioral changes or due to the presence of an obstacle and/or uneven ground conditions. One example is a situation where an individual suddenly tries to reach an object on the right side of his/her body (Fig. 12A). There will be one possible solution. If experiments of the two-joint link model would have been carried out by changing the angles that the original studies have set up (Fig. 3; Fujikawa et al. 1996; Fujikawa et al. 1997; Oshima et al. 1999; Oshima 2008), some of the questions might have been experimentally addressed and solved. As illustrated in Fig. 3, the original experiments of the two-joint link model have set up defined angles for the limb joints, θ_1_, θ_2_ , and θ_3_, and have been carried out only at fixed angles in both the sagittal plane and the transverse plane for forelimb and in the sagittal plane for hindlimb (Fig. 3). However, independent changes of these angles might create a series of conditions for directional changes in forelimb movements in the transverse plane, mimicking a variety of sudden shifts of forelimb directions, since the original experiments for forelimbs were carried out in the transverse plane (Fig. 3B). The similar experiment should be done for hindlimbs in the transverse plane.

For humanoid bi-pedal and quadrupedal robotics, several computational and/or mechanical approaches with or without a passive control of robotic body posture, that is, an enumeration and selection of contact configurations of robotic feet, a development of 3D actuated Dual-SLIP model for robotic legs, and a passivity-based robotic whole-body controller, respectively, have been implemented to control movements of robotic limbs and/or the body in uneven ground conditions (Moraud et al. 2008; Liu et al. 2016; Mesesan et al. 2019). Note, however, that the robots that these studies have used all had an actuator in each joint without any actuators crossing two joints. Therefore, none of these studies facilitates us to formulate a working hypothesis of functional roles of mono- and bi-articular muscles in the two-joint link model under uneven ground conditions.

Considering the contribution of mono- and bi-articular muscles to proposed contact task and torque transfer function of the muscles in the thigh (Fujikawa et al. 2001; Kanayama et al. 2001; Oshima et al. 2004; Oshima et al. 2005; Fujikawa and Oshima 2010; Oshima et al. 2017), Biewener and Daley (2007) and Voloshina et al. (2013) have provided us with a clue for making a testable hypothesis of functional roles of mono- and bi-articular muscles in irregular conditions. Biewener and Daley (2007) discussed regional differences between the proximal muscles of the thigh and the distal “muscle-tendon architecture” of hindlimbs and different responses between two segments of limbs to modulatory changes from the ground. According to their discussion, the proximal muscles including mono- and bi-articular muscles in the thigh do mechanically respond under a “feedforward” control whereas the distal “muscle-tendon architecture” does respond in such an intrinsic manner that the architecture dumps but distributes efficiently effects of a sudden change and/or disturbance to the joints and muscles of the limb.

Upon the human bi-pedal walking on the smooth surface or uneven surface, Voloshina et al. (2013) showed that the positive work load of the hip joint increased substantially on the uneven surface in a less energy efficient manner, suggesting to them that the work load of the hip joint might have been mechanical on the uneven surface. When a human walks in a normal gate and enters the initial stage of the stance phase, his or her heel touches the ground and begins to encounter the ground reaction force. As proposed in Fig. 10, a combined force of *Fe3* and *Fe2* exert its output force and thus direction of the force when e1 and f1 switch their activities. At the foot flat and heel off stages, *Ff1* along with *Fe2* to *Ff2* exerts the output force from the switch AB to BC. Here, the first and second ground reaction forces will be loaded on her or his foot to heel sequentially (Fig. 10). This f1 series muscles will be the gluteus maximus and/or gluteus medius, the action that would stabilize the pelvis on the transverse plane (Afschrift et al. 2018). Therefore, even on the smooth surface, the hip joint becomes mechanically active and these muscles may play a role in balancing the body against different ground reaction forces. The action of the muscles in the hip joint might thus be regarded as the mechanical work upon a normal loading.

By knowing differences in biomechanical properties and functional roles but closely integrated interactions between the proximal muscles and the distal “muscle-tendon architecture” in hindlimbs (Biewener and Daley 2007; Voloshina et al. 2013), Widajewicz et al. (1994), McFadyen et al. (1999) and Chu et al. (2017) highlight our further discussion on possible functional roles of mono- and bi-articular muscles in hindlimbs upon a sudden change in orientation and/or irregular behavioral movements. These studies investigated the cat quadrupedal walking gait focusing on the hindlimbs when cats were subjected to walking with and without obstacles.

Widajewicz et al. (1994) examined the activities of the motor cortex and several limb muscles of hindlimbs along with forelimbs when cats were subjected to walking without obstacles or walking and stepping over obstacles on a moving treadmill. When the cats stepped over an obstacle, the hyper-flexion of the knee, the extension of all joints with the dorsiflexion of the digits over the obstacle, the flexion of the hip and then touch-down on the ground have sequentially occurred (McFadyen et al. 1999; Chu et al. 2017). These activities occurred at the initial stage of the swing phase when, for instance, the right hindlimb is flexed, the action that brings the limb forward (Fig. 12B). In the normal quadrupedal walking gait, according to McKay et al. (2007), Frigon et al. (2015), and Markin et al. (2012), the iliopsoas (a mono-articular muscle) (e1), the medial sartorius (a bi-articular muscle) (e3), the semitendinosus (a bi-articular muscle), and posterior biceps (a bi-articular muscle) (f3) and the tibialis anterior (Ta) all become active. The semitendinosus and posterior biceps (f3) are inactivated immediately after the initial stage of the swing phase. Prior to the end of the swing phase when the right hindlimb touches the ground, the iliopsoas (e1), medial sartorius (e3), and tibialis anterior (Ta) cease their activities whereas the vastus (a mono-articular muscle) (e2) and the gastrocnemius (Gs) become active at the end of the swing phase. Based on the onset and offset of these muscles, we predict that in the light of the two-joint link model, the activity switch in the initial stage of the swing phase would be in the transition from CD (output forces *Ff3* and *Ff2*) to DE (output forces *Ff2* and *Fe1*) (Figs. 5 and 12B). In Fig. 12, three axes, AD, BE, and CF, are illustrated in the stride images of the normal gait and the obstacle avoidance gait along with predicted output forces in the switches CD and DE.

When a cat tries to avoid an obstacle in the initial stage of the swing phase, the knee of the right hindlimb is hyper-flexed and then the hindlimb becomes extended immediately forward as the hip joint is flexed. According to Widajewicz et al. (1994), the action of this avoidance hyper-activates the semitendinosus (f3) and tibialis anterior and activates the gastrocnemius, and then immediately hyper-activates the medial sartorius (e3) and vastus (e2) (Fig. 12B). Except the gastrocnemius, the activation pattern is identical to that of the normal waling gait, but all the muscles are hyper-activated to avoid the obstacle. The vastus (e2) might have been activated earlier than in the normal quadrupedal walking gait due to the hyperflexion of the knee. Illustrated three axes and output forces in CD and DE ranges indicate that the forces exerted, directions obtained, and angles in the right hindlimb during the obstacle avoidance gait differ from these in the normal gait, suggesting that mono- and bi-articular muscles might modulate their activities in response to the avoidance of the obstacle (Fig. 12B). Even, as suggested by several studies (Latash 2018; Saliba et al. 2020; Munoz-Martel et al. 2021), a sudden avoidance of the obstacle might activate simultaneously an antagonistic pair of muscles (e3 and f3) and increase the stiffness of the hindlimb. As discussed above, therefore, by constructing stride images with three axes, measuring and possibly calculating strengths of output forces and actual directions of forces, stimulating captured images with kinetic data, advanced EMGs, for example, high-density EMG (Watanabe et al. 2021), motion capture system, a force plate, force sensors, three-dimensional analyses, and/or computerized analyses all facilitate to make a comparison and to generate a testable hypothesis of biomechanics of a normal and an obstacle avoidance gait in light of the two-joint link model.

### Digitigrade and unguligrade locomotion

Humans are plantigrade and walk and run on the entire foot distal to the heel. In contrast, some of tetrapods including birds, dinosaurs, cats, dogs, pigs, hippopotamuses, and elephants are digitigrade and walk and run on their phalanges rather than the heel and proximal metatarsals. Other tetrapods including horses and cattle are unguligrade and walk and run on the most distal phalanx (Carrano 1997). As the two-joint link model proposes that a different combination of mono- and bi-articular muscles in the human thigh exerts its output force at the ankle, whether these muscles in digitigrade and unguligrade tetrapods would exert an output force at the ankle or at a more distal joint remains to be tested (Fig. 12C, D).

Oshima et al. (2004) and Oshima et al. (2005) have shown that mono- and bi-articular muscles of robotic leg models are able to control contact task and maintain the posture with the torque transfer function from the proximal to distal joints. Thus, as digitigrade cats have a muscle pattern of the thigh and lower leg identical to that of humans, Oshima et al. (2010) proposed that digitigrade animals, cats and dogs, have output forces from mono- and bi-articular muscles at the ankle, being identical to humans due to the torque transfer function of these muscles to the ankle (Fig. 12C). Unfortunately, since these studies have been performed solely as mechanical and robotic experiments, whether the torque transfer function actually would occur in digitigrade animals remains to be examined and tested.

However, unlike digitigrade cats and dogs which have the tibialis anterior as a mono-articular muscle on the dorsal side and the gastrocnemius as a bi-articular muscle on the ventral side of the lower leg, unguligrade horses have a set of bi-articular muscles, the fibularis tertius (Ft) (peroneus tertius) on the dorsal side and the gastrocnemius on the ventral side (Fig. 12D). The fibularis tertius originates from the extensor fossa of the lateral condyle on the femur and inserts on the third metatarsal bone (Liebich et al. 2014) (Fig. 12D). In addition, the fibularis tertius and the gastrocnemius have been regarded as the reciprocal apparatus in horses to move jointly the knee joint and the hock (ankle) joint (Wentink 1978 and 1979; Maierl et al. 2014). Therefore, the existence of two sets of bi-articular muscles in the horse thigh and lower leg represents what van Ingen Schenau (1990) proposed as a co-activation of bi-articular muscles in two consecutive linked segments to increase the net joint power in the hip joint and the knee joint (Fig. 1B).

The question is then how two sets of bi-articular muscles work at two segments with regard to their output forces (Fig. 12D). There will be two different conditions: the bi-articular and mono-articular muscles in the thigh exert their forces either at the hock (ankle) joint or at a more distal joint, the fetlock joint (between the third metatarsal and proximal phalanx), the pastern joint (between the proximal phalanx and middle phalanx), or the coffin joint (between the middle phalanx and distal phalanx) (Piché et al. 2020; Fig. 12D). If two sets of bi-articular muscles interact, how would this differ from the condition described above with a single set of interacting bi-articular muscles? Do the two sets of bi-articular muscles exert their forces independently? How are their activities coordinated to control the movement of the entire limb?

McGuigan et al. (2009) examined the muscle-tendon of the gastrocnemius and the superficial digital flexor, a thin muscular tissue on the ventral side of the lower leg, during steady and unsteady locomotive activities and suggested that their output work changes in response to the different activities. As discussed by Biewener and Daley (2007), there could thus be an energy transfer function from the proximal muscles and tendons, probably the bi-articular muscles of the thigh, to the distal muscles including the gastrocnemius and superficial digital flexor (Elftman 1939). By examining the muscles of horse hindlimbs, Payne et al. (2005) showed that the muscles associated with the proximal hindlimb were larger in volume but had shorter fascicles as compared with those in the lower hindlimb and concluded that the proximal muscles tended to exert large forces but that the distal ones including the tendons might serve to store elastic energy. Furthermore, the tendons and joints of the distal hindlimbs have been shown to facilitate storage of elastic energy (Biewener 1998; Harrison et al. 2010), but they also experienced the highest loadings (Harrison 2010).

Furthermore, as discussed above, Biewener and Daley (2007) made a discussion on the integration of muscle functions with postural changes and neuromuscular control for unsteady locomotion. In fact, their discussion is not limited to the locomotion in unsteady ground conditions but can be applicable to the locomotion in normal ground conditions. The important issues of digitigrade and unguligrade locomotion in light of the two-joint link model are thus (1) dynamic but regional differences between the proximal muscles of the brachium or thigh and the distal “muscle-tendon architecture” of forelimbs or hindlimbs (Biewener and Daley 2007); (2) dynamic interplays and responses between the proximal muscles and the “muscle-tendon architecture” to any modulatory mechanical loadings from the ground (Biewener and Daley 2007); (3) net-output forces and directions of the limbs as a result of interactions between the proximal and distal segments with or without postural changes (Biewener and Daley 2007). When Biswal and Mohanty (2021) reviewed the development of quadrupedal walking robots, they expressed their opinion that including cats, cheetahs and dogs, toed animals are mechanically able to run faster with higher energy efficiency than non-toed animals.

Larger fascicles and elaborate tendon architecture in horse hindlimbs might constitute, thereby, a further specialized locomotive apparatus for receiving large output forces (Payne et al. 2005) and extended range of stiffness (Höppner et al. 2014) from the upper hindlimb, storing this elastic energy (Biewener 1998; Harrison et al. 2010) and counteracting high loads against the ground (Harrison et al. 2010). After Wentink (1978) examined the reciprocal apparatus of the lower hindlimbs in seven horses for a quadrupedal walking gait investigation, he concluded that the fibularis tertius and the gastrocnemius might not be involved in the movements of the hindlimb; instead, they “may centre the force of the load through the long axis of the tibia” and that the tendons of the distal lower hindlimbs stored elastic energy. In fact, the co-activation of the bi-articular muscles, the fibularis tertius (Ft) and the gastrocnemius (Gs), might increase the stiffness in the distal joints (Latash 2018: Fig. 12D). Regardless of whether the e- and f-series of the muscles in the horse thigh exert their forces at the hock or at a more distal joint of the hindlimb, the distal architecture of the joints, muscles, and tendons in horse hindlimbs might have an important role in receiving large forces from the proximal muscles, storing elastic energy, and/or withstanding loads from the ground. The integration of the two-joint link model into further investigations will, therefore, be essential for understanding the structure and function of horse hindlimbs and forelimbs, both limbs which have a “load and resistance factor” design.

### The motor control of mono- and bi-articular muscles

Since the two-joint link model, as established originally using EMG data, has been mechanically tested with robotic arms and legs, the motor control of the arms and legs was mechanical, dynamic, rapid acting, and/or preflexive (Fujikawa 2016). One single feedforward command was able to activate the arms and legs equipped with two pairs of mono-articular actuators and one pair of bi-articular actuators (Oshima et al. 2000; Oshima et al. 2001). The actuators used for the studies include pneumatic artificial rubber actuators based on viscoelastic muscle model (Fujikawa et al. 1996; Oshima et al. 1999; Oshima et al. 2001), compression springs (Fujikawa et al. 2001; Oshima et al. 2004; Oshima et al. 2005; Fujikawa et al. 2010; Fujikawa and Oshima 2010; Fujikawa 2016; Oshima et al. 2017), wires (Oshima et al. 2004; Oshima et al. 2005) or servomotors (Oshima et al. 2017) or a combination of these actuators (Oshima et al. 2017).

Therefore, all the studies took a serious consideration of material properties of the actuators for robotic testing in order to emulate biological properties of muscles and tendons. These studies suggest the following biomechanical properties of coordinated activities of mono- and bi-articular muscles in limbs: (1) exerted output forces and directions by these muscles at the wrist or ankle, not the elbow or knee; (2) a single feedforward command that enables an activation of a different pair of mono- and bi-articular muscles; (3) a mechanical feedback(s) that has been observed potentially as energy transfer from a compression spring of the leg segment to the thigh segment when robotic legs engaged jumping experiments.

Despite the efforts, the studies of the model did not provide us with insights of biological motor control of coordinated activities of mono- and bi-articular muscles in limbs and interplays between the proximal and distal segments of limbs. Recent neurobiological studies might have clues for motor neuronal circuits for limb locomotion that are composed of the motor cortex, basal ganglia, brain stem, and the spinal cord where the central pattern generators (CPGs) and lateral motor columns (LMCs) are located. Grillner and Manira (2020) described different components of these neural parts, different signal pathways including feedforward and feedback signal pathways among the central nervous system, spinal cord, visual system, vestibular system, and proprioception in tendons, muscles, and limbs.

The conceptual framework and data that the neurobiological studies have generated might thus enable us to predict a possible simple model for motor control of mono- and bi-articular muscles in limbs. First of all, the motor neurons innervating the extensor and flexor muscles of both the hindlimbs and forelimbs lie in a specific region of the spinal cord known as LMCs (VanderHorst and Holstege 1997; Sürmeli et al. 2011; Machado et al. 2015; Gross et al. 2017; Jung et al. 2018). The motor neurons on the lateral side of the ventral horn in the spinal cord innervate the extensor muscle, whereas those on the medial side innervate the flexor muscle. In contrast, this relationship is reversed in forelimbs: motor neurons on the lateral side of the ventral horn in the spinal cord innervate the flexor muscle whereas those on the medial side innervate the extensor muscle (Pearson and Gordo 2013). VanderHorst and Holstege (1997) mapped the motor neuronal cells in the spinal cord of cats that innervate the hindlimb muscles (iliopsoas (e1), psoas major (e1), sartorius, adductor magnus, adductor brevis, pectineus, gracilis, vastus lateralis (e2), vastus medialis (e2), rectus femoris (e3), gluteus maximus (f1), gluteus medius (f1), tensor fasciae latae, biceps femoris (f3), semimembranosus (f3), semitendinosus (f3), gastrocnemius (Gs), soleus and tibialis anterior (Ta) as well as the pelvic floor, axial and foot muscles). Sürmeli et al. (2011) and Gross et al. (2017) refined the technique of VanderHorst and Holstege (1997) investigated and mapped the motor neurons in mice and in monkeys and humans, respectively. Both works show the medio-lateral organization but also dorso-ventral organization of the motor pools in the ventral horn of LMC, each of which relates to a specific muscle on the dorsal side or ventral side and to proximo-distal regions of the pelvic girdle and hindlimb. These findings suggest to us that we might be able to predict potential motor neuronal circuits in LMCs that would generate and control coordinated activities of a different pair of mono- and bi-articular muscles during a gait cycle (Fig. 12E).

There are two LMCs in the spinal cord, the branchial LMC (b-LMC) for forelimbs and the lumbar LMC (l-LMC) for hindlimbs in mammals including cats, rats, mice, and humans examined (Murakami and Tanaka 2011). According to the two-joint link model, the e-series muscles are located on the dorsal side of both limbs whereas the f-series muscles are located on the ventral side of both limbs (Fig. 2A). Both the e- and f-series muscles extend and flex the entire hindlimb or forelimb. Based on the neurobiological studies (VanderHorst and Holstege 1997; Sürmeli et al. 2011; Machado et al. 2015; Gross et al. 2017), we predict that there is the medio-lateral and dorso-ventral segregation of the neurons in motor pools of the LMCs that innervate the bi- and mono-articular muscles in the hindlimb and forelimb. In addition, we set up the locations, medial (M) and lateral (L), in the LMCs and activation sequences of the motor neurons based on the two-joint link model (Fig. 12E). The neurons that innervate the e-series muscles and f-series muscles in forelimbs are located on the medial side (M) of the b-LMC and on the lateral side (L) of the b-LMC, respectively, (Agbarajl et al. 2017; Fig. 12E). On the other hand, those that innervate the e-series muscles and tibialis anterior (Ta) and the f-series muscles and gastrocnemius (Gs) in hindlimbs are located on the lateral side (L) of the l-LMC and on the medial side (M) of the l-LMC, respectively, (Fig. 12E; VanderHorst and Holstege 1997; Sürmeli et al. 2011). The neurons that innervate e1/f1 pair, e3/f3 pair, or e2/f2 pair might be located on the most rostro-ventral section of the b-LMC and the l-LMC (Fig. 12E, position 4), on the mid-section of the b-LMC and the l-LMC (Fig. 12E, position 3) and on the caudo-dorsal section of the b-LMC and the l-LMC (Fig. 12E, between position 2 and 3), respectively. The neurons that innervate the tibialis anterior (Ta) and gastrocnemius (Gs) as well as the muscles associated with the foot are located on the most caudo-dorsal section (Fig. 12E, position 2). The motor pool innervating the muscles associated with the foot is located at the most dorso-caudal section of the l-LMC. However, according to the studies, this section of the motor pool does not appear to be segregated into the lateral and medial sides (Fig. 12E; VanderHorst and Holstege 1997; Sürmeli et al. 2011, position 1).

As have previously studied, the motor neuronal circuits of the b-LMC and the l-LMC associated with the sequential activations of motor neurons in light of the two-joint link model are thus predicted to involve interactive neural circuits of the CPGs, interneurons, motor neurons within the b-LMC or the l-LMC and between the b-LMC and the l-LMC and sensory neurons (Jessell 2000; Rybak et al. 2015; Côté et al. 2018; D'Elia and Dansen 2018; Osseward and Pfaff 2019; Grillner and Manira 2020). Therefore, the motor neural circuits that we predict might have specific characteristics of activation patterns and underscore the need for experimental and/or simulation analyses to advance further the framework that have been conceptualized on the basis of the two-joint link model.

### Feedback modulations of motor control of mono- and bi-articular muscles

The mechanical studies of the two-joint link model of mono- and bi-articular muscles have shown that a different combination of mono- and bi-articular muscles exerts a combined output force at the wrist or at the ankle where the force would counteract the ground reaction forces (Figs. 7 and 10; Fujikawa et al. 2001; Kanayama et al. 2001; Oshima et al. 2004; Oshima et al. 2005; Fujikawa and Oshima 2010; Oshima et al. 2017). However, considering the anatomical and functional complexity of limb architecture, especially, hands and feet, the model especially focusing on contact task alone fell far short of unraveling biologically dynamic interplays between the proximal muscles and the “muscle-tendon architecture,” that is, involvements of mechanical and sensory feedbacks between the proximal and distal segments of limbs. For instance, the human “muscle-tendon architecture” of hindlimb consists of the leg and foot where there are two bones in the leg and totally 26 bones including seven tarsal bones, 5 metatarsals, and 14 phalanges in the foot with elaborated ligamentous networks. Between the leg and foot, four muscles including the tibialis anterior on the dorsal side and nine muscles including the gastrocnemius and soleus on the ventral side are organized with their extensive tendons into the foot. In the foot, 13 muscles are associated as the foot intrinsic muscles with 26 bones along with the aponeurosis, ligaments and adipose tissues on the plantar surface (Schünke et al. 2017; Martini et al. 2018).

With this complex architecture, the foot plays multiple functional roles in bearing body weight and thus distributing forces during weight bearing, coordinating mechanical motions of the body and upper hindlimb and ground reaction forces. (Elftman 1969; McPoil and Knecht 1985; Chan and Rudins 1994; Brockett and Chapman 2016). In particular, the ankle makes different motions during bi-pedal walking: (1) in plantarflexed position where the subtalar joint is everted and abducted, thus pronated, when the heel strikes the ground at the beginning of the stance phase. Then, the foot touches the ground; (2) from plantarflexed position to dorsiflexed position where the subtalar joint is inverted and adducted, thus supinated, in the mid stance phase. The dorsal side of the leg moves forward around the ankle and the body moves forward; (3) to plantarflexed position when the toe leaves off the ground in the terminal stance phase (McPoil and Knecht 1985; Chan and Rudins 1994; Brochett and Chapman 2016). The power of the ankle joint becomes maximum when the single hindlimb supports the entire body in the mid stance phase, approximately 50% of the gait cycle (Brockett and Chapman 2016). The peak of the ground reaction force is, however, at the transition between the time when the body weight is transferred to the forward limb and mid stance (Perry and Burnfield 2010).

Therefore, the cyclic bi-pedal walking results in repetitive mechanical motions in different parts of the foot and thus the stress to the foot due to different strengths and rates of loadings including the ground reaction force. However, once normal gait pattern is disrupted, for instance, on uneven ground surface, the foot as well as leg would detect external and internal cues and regain normal patterns of movement and/or gait. Proprioception has been considered essential to detect these cues and control the movement of the body since Sherrington (1906) coined the term. The structures of musculoskeletal system including muscles, tendons, ligaments, joints, cartilages and bones, connective tissues, and the skin of the body all have known to possess receptors in proprioception, engage mechanosensory detections, and make sensory feedbacks to the central nervous system (Landelle et al. 2021; Moon et al. 2021). However, as regarded to be the sixth sense system, cellular, and molecular characteristics of mechanosensory receptors in proprioception have long remained uncertain until two genes encoding for mechanosensory ion channels, *Piezo1*, and later *Piezo2*, on cell membranes have been cloned and characterized (Coste et al. 2010; Ranade et al. 2014; Woo et al. 2015).

*Piezo1 and Piezo 2* are now known to be expressed as the mechanosensory ion channel on cell membranes of a variety of tissues and organs including the structures of the musculoskeletal system, according to The Human Protein Atlas Project (funded by the Knut & Alice Wallenberg foundation) (https://www.proteinatlas.org/ENSG00000154864-PIEZO2). Therefore, a number of experimental studies have demonstrated that both genes are responsible for detecting mechanical pressure and thus sensing body posture and motion (Bornstein et al. 2021). One of the studies was to experimentally demonstrate the role of *a variant Piezo1*, E756del *Piezo1*, found in some of West Africans, African Americans, and Europeans (Nakamichi et al. 2022). The cohort study indicates that this variant was present in Jamaican sprinters at higher frequency than non-athletic Jamaicans (Nakamichi et al. 2022). When the variant was experimentally induced into mouse tendons, the mice with this variant had higher jumping and faster running ability than control mice. All results suggest a functional improvement of the tendons of the mice with the induced variant and its natural functional association with “enhanced tendon anabolism” (Nakamichi et al. 2022). What we gain from an advancement in molecular and cellular studies is to expand our scientific and experimental repertoires for elucidating underlying mechanisms of dynamic interplays between the proximal muscles and the “muscle-tendon architecture,” modulations of limb locomotion through mechanosensory feedbacks in proprioception coordinated with other sensory systems and interactions between and/or among the structures of the musculoskeletal system in any parts of limbs.

As the “muscle-tendon architecture,” therefore, the leg and, in particular, the foot including the ankle joint might be a key anatomical and functional structure to play its pivotal roles in controlling and/or modulating limb locomotion. As Biewener and Daley (2007) discussed, when forces are exerted on the foot by mono- and bi-articular muscles in the thigh, the foot encounters physical stimuli from environments, responds the effects, feedbacks the information of the effects to the other parts of the limb and thus acts as the key modulator for limb locomotion. We no longer treat the limb musculature merely as extensor or flexor muscles for neural motor control analysis, since different segments of limbs, for example, the thigh, leg, foot, have a distinct set of the musculoskeletal structures with their unique dynamic feedforward and feedback systems and because a given segment would interact differently with other segments. Our challenge is, therefore, to integrate structures, movements, motor control, networks of feedforward, and feedback signals and regulatory control within and among different biological structures along with physical stimuli into a testable hypothesis for tetrapod limb locomotion. As described and discussed in our present review, proper assessments of biological parameters gleaned from mechanically tested two-joint link model and establishment of testable hypothesis based on biological data will hopefully facilitate us to make solutions for unsolved issues that we have discussed in the present review.

## Conclusions

Our present review of the two-joint link model and the examples of its application for studies of tetrapod locomotion will hopefully pave the way for incorporating the role of coordinated activities of bi- and mono-articular muscles in limbs into future studies of tetrapod limb locomotion. Serving as a foundation for these are earlier studies on the functional and mechanical link between the proximal and distal elements of limbs (Ellerby and Marsh 2010; Desrochers et al. 2018; Schumacher et al. 2020). Since Dutch and Japanese groups have focused their studies on bi-articular muscles in human limbs, recent studies of limb locomotion focusing on bi-articular muscles have been mechanical and/or robotic, in particular, for constructions of humanoid robots (Ogawa et al. 2011; Lewis et al. 2011; Markowitz and Herr 2016; Garcia-Rodriguez et al. 2020). However, there has been only little research on functional roles of bi-articular muscles and their coordinated roles with mono-articular muscles. Notable exceptions are a study of biomechanical template models based on the spectrum of locomotor subfunctions (Schumacher et al. 2020) and another study using high-density surface EMG to examine regional activation of bi-articular muscles in humans (Watanabe et al. 2021).

Given the recent advances in development of motion capture systems, a force plate, force sensors, EMG techniques including high-density surface EMG, three-dimensional, and/or computerized analyses (Oshima 2008; Karakasiliotis et al. 2016; Fischer et al. 2018; Seth et al. 2018; Manafzadeh et al. 2021; Watanabe et al. 2021; Gatesy et al. 2022), the two-joint link model could potentially be incorporated into and/or tested against different models and/or modeling of tetrapod locomotion, including biomechanical template model (Sharbafi et al. 2017; Sharbafi et al. 2018; Schumacher et al. 2019; Schumacher et al. 2020), joint torque models (Geyer and Herr 2010; Madeti et al. 2014; Agbarajl et al. 2017), joint coordinate system (Pereira et al. 2012; Baier et al. 2013; Lin et al. 2019; Gatesy et al. 2022), synergy hypothesis (Bernstein 1967; Desrochers et al. 2018; Singh et al. 2018), and musculoskeletal modeling (Hutchinson et al. 2015; Seth et al. 2018; Bishop et al. 2021; De Groote and Falisse 2021). Some of models focuses on a single joint or multiple joints, some focuses on the entire musculoskeletal system or some focuses on synergistic muscle activities regardless of a number of joints. On the other hand, the two-joint link model of mono- and bi-articular muscles in forelimbs and hindlimbs has had its different focus by investigating coordinated activities of the muscles between two joints but their output forces and directions in the distal joints. Thus, contributions of the two-joint link model to the biomechanics of tetrapod limb locomotion might be limited to some degree, but outcomes of the model would be testable against any other models. In our present review, we have extracted biological parameters and underlying concept from mechanically tested model of limb locomotion equipped with mono- and bi-articular muscles and will hopefully be able to test tetrapod limb locomotion from a biological point of view. Hopefully, our present review will contribute to further the understanding of robotics-inspired biology and biomechanics (Gravish and Lauder 2018).
